# Nucleolar GTPase Bms1 displaces Ttf1 from RFB-sites to balance progression of rDNA transcription and replication

**DOI:** 10.1093/jmcb/mjab074

**Published:** 2021-11-13

**Authors:** Yanqing Zhu, Yong Wang, Boxiang Tao, Jinhua Han, Hong Chen, Qinfang Zhu, Ling Huang, Yinan He, Jian Hong, Yunqin Li, Jun Chen, Jun Huang, Li Jan Lo, Jinrong Peng

**Affiliations:** MOE Key Laboratory for Molecular Animal Nutrition, College of Animal Sciences, Zhejiang University, Hangzhou 310058, China; Taizhou Hospital, Zhejiang University, Taizhou 317000, China; MOE Key Laboratory for Molecular Animal Nutrition, College of Animal Sciences, Zhejiang University, Hangzhou 310058, China; The MOE Key Laboratory of Biosystems Homeostasis and Protection and Innovation Center for Cell Signaling Network, Life Sciences Institute, Zhejiang University, Hangzhou 310058, China; MOE Key Laboratory for Molecular Animal Nutrition, College of Animal Sciences, Zhejiang University, Hangzhou 310058, China; MOE Key Laboratory for Molecular Animal Nutrition, College of Animal Sciences, Zhejiang University, Hangzhou 310058, China; MOE Key Laboratory for Molecular Animal Nutrition, College of Animal Sciences, Zhejiang University, Hangzhou 310058, China; MOE Key Laboratory for Molecular Animal Nutrition, College of Animal Sciences, Zhejiang University, Hangzhou 310058, China; Institute of Biotechnology, Zhejiang University, Hangzhou 310058, China; Institute of Biotechnology, Zhejiang University, Hangzhou 310058, China; College of Life Sciences, Zhejiang University, Hangzhou 310058, China; The MOE Key Laboratory of Biosystems Homeostasis and Protection and Innovation Center for Cell Signaling Network, Life Sciences Institute, Zhejiang University, Hangzhou 310058, China; MOE Key Laboratory for Molecular Animal Nutrition, College of Animal Sciences, Zhejiang University, Hangzhou 310058, China; MOE Key Laboratory for Molecular Animal Nutrition, College of Animal Sciences, Zhejiang University, Hangzhou 310058, China

**Keywords:** Bms1, cell cycle, nucleolus, ribosome small subunit processome, replication-fork barrier, Ttf1, zebrafish

## Abstract

18S, 5.8S, and 28S ribosomal RNAs (rRNAs) are cotranscribed as a pre-ribosomal RNA (pre-rRNA) from the rDNA by RNA polymerase I whose activity is vigorous during the S-phase, leading to a conflict with rDNA replication. This conflict is resolved partly by replication-fork-barrier (RFB)-sites sequences located downstream of the rDNA and RFB-binding proteins such as Ttf1. However, how Ttf1 is displaced from RFB-sites to allow replication fork progression remains elusive. Here, we reported that loss-of-function of Bms1l, a nucleolar GTPase, upregulates rDNA transcription, causes replication-fork stall, and arrests cell cycle at the S-to-G2 transition; however, the G1-to-S transition is constitutively active characterized by persisting DNA synthesis. Concomitantly, *ubf*, *tif-IA*, and *taf1b* marking rDNA transcription, Chk2, Rad51, and p53 marking DNA-damage response, and Rpa2, PCNA, Fen1, and Ttf1 marking replication fork stall are all highly elevated in *bms1l* mutants. We found that Bms1 interacts with Ttf1 in addition to Rc1l. Finally, we identified RFB-sites for zebrafish Ttf1 through chromatin immunoprecipitation sequencing and showed that Bms1 disassociates the Ttf1‒RFB complex with its GTPase activity. We propose that Bms1 functions to balance rDNA transcription and replication at the S-phase through interaction with Rcl1 and Ttf1, respectively. TTF1 and Bms1 together might impose an S-phase checkpoint at the rDNA loci.

## Introduction

During the S-phase, eukaryotic pre-ribosomal RNA (pre-rRNA), the precursor for 18S, 5.8S, and 28S ribosomal RNAs (rRNAs), is actively synthesized by RNA polymerase I (Pol-I), leading to either a head-on or codirectional conflict between rDNA transcription and replication (Klein and Grummt, 1999; Akamatsu and Kobayashi, 2015). The head-on conflict is resolved by the replication fork barriers (RFBs) containing DNA sequences downstream of the RNA gene, whereas the codirectional conflict is resolved by the RFBs formed by an RNA‒DNA hybrid called R-loop (Gerber et al., 1997; Kobayashi, 2003; Mirkin and Mirkin, 2007; Akamatsu and Kobayashi, 2015; Hamperl and Cimprich, 2016). In baker’s yeast, the locus of rDNA contains ~150 tandem repeats of the 35S pre-rRNA genes separated by 5S rRNA gene (Henras et al., 2015) and the head-on RFBs are localized between the 35S pre-rRNA and 5S rRNA genes (Linskens and Huberman, 1988). The head-on RFB sequences serve as the docking site for protein factors such as Fob1 to resolve the collision between transcription and replication (Kobayashi, 2003; Mirkin and Mirkin, 2007; Akamatsu and Kobayashi, 2015; Hamperl and Cimprich, 2016). In mouse and human cells, the head-on RFB sequences, also called the ‘Sal-boxes’, are located downstream from the 47S pre-rRNA-coding region (Bartsch et al., 1987; Lopez-estrano et al., 1998; Akamatsu and Kobayashi, 2015) and Ttf1 is the factor to bind to the head-on RFB-sites to terminate rDNA transcription and mediate replication fork arrest (Bartsch et al., 1987; Evers et al., 1995; Diermeier et al., 2013; Akamatsu and Kobayashi, 2015). However, how TTF1 is displaced from the head-on RFB-sites to allow the replication fork progression remains elusive.

Nucleolus harbors machineries responsible for processing and maturation of 18S, 5.8S, and 28S rRNAs from the pre-rRNA and assembly of ribosomal small and large subunits (Boisvert et al., 2007; Henras et al., 2015). Compromised ribosome biogenesis might cause related diseases named ribosomopathies (Farley-Barnes et al., 2019; Huang et al., 2020). As a component of the small subunit (SSU) processome (Pérez-Fernández et al., 2011; Phipps et al., 2011), Bms1, a nucleolar GTPase (Karbstein et al., 2005; Karbstein and Doudna, 2006), partners with Rcl1 to cleave pre-rRNA at specific sites (Billy et al., 2000; Wegierski et al., 2001; Delprato et al., 2014; Kornprobst et al., 2016; Cheng et al., 2017; Zhu et al., 2021). Interestingly, studies have shown that mutation in zebrafish Bms1l displays a hypoplastic liver (Wang et al., 2012) and malfunctioned BMS1 in human causes aplasia cutis congenital (Marneros, 2013), both due to cell cycle arrest. However, how Bms1l/BMS1 controls cell cycle progression is currently unknown.

Successful completion of cell cycle is controlled by the G1/S, S/G2, G2/M, and metaphase-to-anaphase transition checkpoints (Keaton, 2007; Malumbres, 2014; Saldivar et al., 2018). Considering the special genomic features of the rDNA loci, which usually harbor hundreds of copies of tandemly arrayed rDNA genes, and the role of TTF1 in resolving the head-on conflict between rDNA transcription and replication, we hypothesized that the on-and-off of TTF1 at the RFB-sites might act as a control of the S-to-G2 progression. We reveal here that nucleolar GTPase Bms1 directly displaces Ttf1 from the RFB-sites to facilitate the replication-fork progression, thus establishing a molecular mechanism for resolving the head-on confliction between transcription and replication at the rDNA loci at the S-phase.

## Results

### Loss-of-function of Bms1l upregulates 45S pre-rRNA transcription and alters nucleolar morphology

The zebrafish *bms1l^sq163/sq163^* homozygous mutant, which harbors L^152^ to Q^152^ substitution in Bms1l, confers a small liver phenotype due to cell cycle arrest, but not to cell apoptosis, characterized by reduced ratios of phospho-Histone H3-positive (pH3-positive) cells (Wang et al., 2012). We generated a new mutant allele *bms1l^zju^*^*1*^ via CRISPR‒Cas9 technology. *bms1l^zju^*^*1*^ carries one base substitution and a 13-base pair insertion in exon2 that disrupts the *bms1l* open-reading frame (Supplementary Figure S1A). Compared with the wild-type (WT) control, the *bms1l^zju1/zju^*^*1*^ homozygotes displayed short trunk, small eyes, heart edema, and hardly detectable signals of *fatty acid binding protein 10a* (*fabp10a*, a liver marker), *intestine fatty acid binding protein* (*ifabp*, an intestine marker), and *trypsin* (an exocrine-pancreas marker) at 5 day-post-fertilization (5dpf) (Supplementary Figure S1B‒E), phenotypes more severe than that observed in the *bms1l^sq163/sq163^* mutant (Wang et al., 2012). Allelism analysis revealed no or a small liver in the *bms1l^sq163/zju^*^*1*^ hemizygotes (Supplementary Figure S1F). Therefore, Bms1l plays an essential role in digestive organ development.

**Figure 1:**
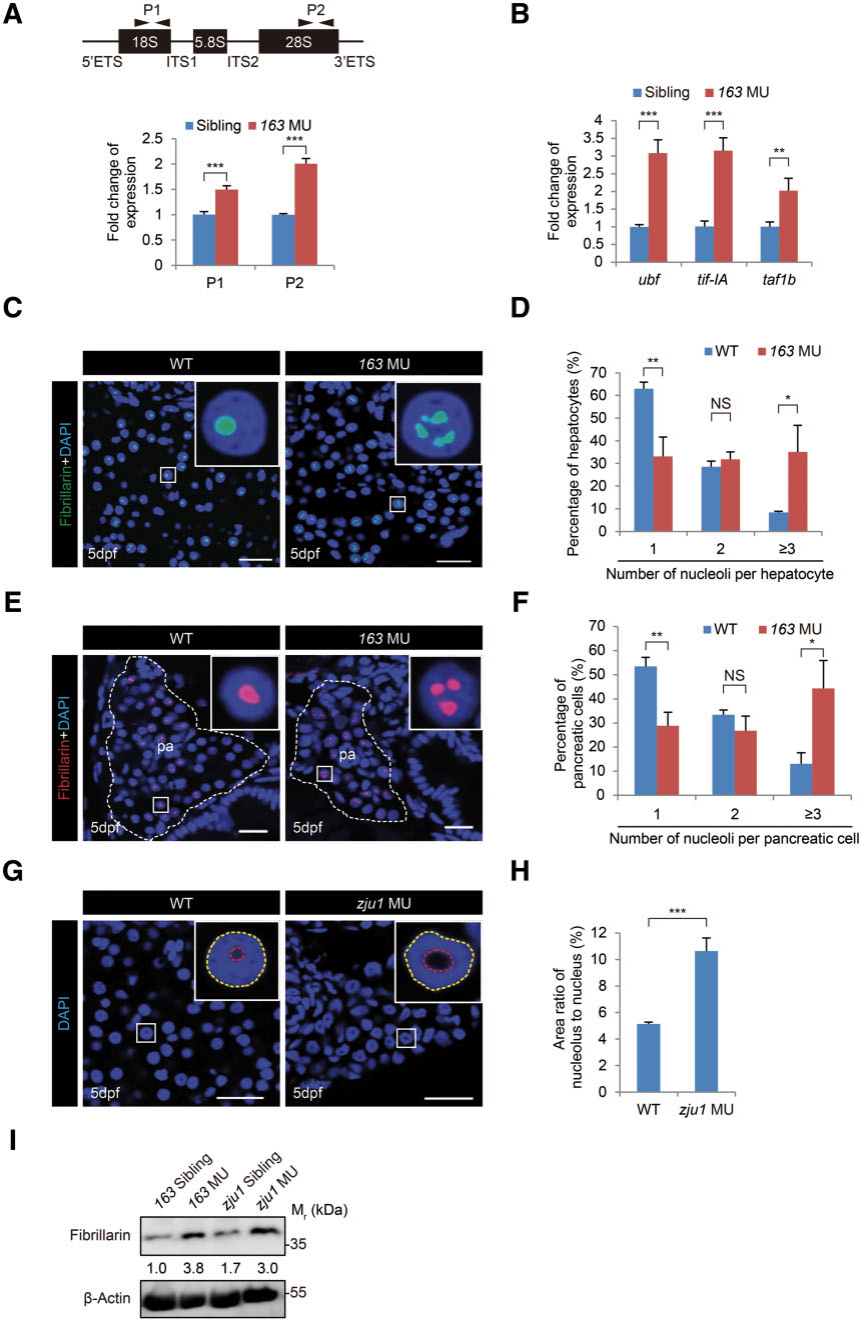
Loss-of-function of Bms1l upregulates the pre-rRNA transcription and increases the volume of nucleoli in hepatocytes. (**A**) qPCR analysis of total rRNA transcripts in 5dpf-old *bms1l^sq163/sq163^* mutant and its siblings (the pool of *bms1^+/+^* and *bms1^sq163/+^*, used thereafter) using two pairs of primers derived from 18S (P1) and 28S (P2) showing an upregulation of rRNA expression in *bms1l^sq163/sq163^* mutant. Upper panel, a diagram showing the genomic structure of the zebrafish rDNA gene and the positions of P1 and P2. (**B**) qPCR analysis showing the upregulation of *ubf*, *tif-IA*, and *taf1b* expression in 5dpf-old *bms1l^sq163/sq163^* mutant compared with the siblings. The qPCR values were normalized against *GAPDH* and expressed as fold change of expression. The values plotted represent mean ± SEM. **P < 0.01, ***P < 0.001. (**C**–**F**) Fibrillarin immunostaining and DAPI staining (green in **C** and red in **E**) showing the significant increase in the number of nucleoli in *bms1l^sq163/sq163^* hepatocytes (**D**, three WT embryos, 1397 cells examined; three *bms1l^sq163/sq163^* embryos, 1274 cells examined) and pancreatic cells (**F**, three WT embryos, 1015 cells examined; three *bms1l^sq163/sq163^* embryos, 463 cells examined) when compared with WT, respectively, at 5dpf. The pancreatic region (pa) was outlined by a dashed line. Scale bar, 20 μm (**C**) and 10 μm (**E**). (**G** and **H**) DAPI staining (**G**) showing the significant increase in the size of nucleolus in *bms1l^zju1/zju1^* hepatocytes compared with WT at 5dpf (**H**, three WT embryos, 112 cells examined; four *bms1l^zju1/zju1^* embryos, 119 cells examined). Scale bar, 20 μm. (**I**) Western blotting showing the upregulation of Fibrillarin protein levels in 5dpf-old *bms1l^sq163/sq163^* and *bms1l^zju1/zju1^* mutants compared with their siblings. β-Actin: loading control. Insets in **C**, **E**, and **G** showing higher magnification of a representative nucleus (boxed). The values in **D**, **F**, and **H** plotted represent mean ± SEM. *P < 0.05, **P < 0.01, ***P < 0.001; NS, no significance.

We previously showed that the pre-rRNA processing was affected after knockdown of Bms1l expression using a *bms1l* gene-specific morpholino (MO) in the WT embryos (Wang et al., 2012). Here, we compared the ratios of 28S rRNA vs. 18S rRNA between *bms1l^sq163/sq163^* and its siblings (the pool of *bms1^+/+^* and *bms1^sq163/+^*) and between *bms1l^zju1/zju^*^*1*^ and its siblings (the pool of *bms1^+/+^* and *bms1^zju1/+^*) at 5dpf, and both *bms1l^sq163/sq163^* and *bms1l^zju1/zju^*^*1*^ homozygous mutants exhibited a much greater 28S vs. 18S ratio than their siblings (Supplementary Figure S2A and B). RNA analysis using the *5ʹ-external-transcribed-spacer* (*5ʹ-ETS*) and *internal-transcribed-spacer-1* (*ITS1*) probes showed an upregulation of the transcript levels of 45S pre-rRNA in both mutants at 5dpf together with two additional bands detected by the *5ʹ-ETS* probe (Supplementary Figure S2C). Quantitative real-time PCR (qPCR) analysis confirmed the upregulation of rRNA expression in *bms1l^sq163/sq163^* at 5dpf (Figure 1A). Concomitantly, genes controlling 45S pre-rRNA transcription including *HMG-box containing upstream binding factor* (*ubf*), *transcription-initiation-factor IA* (*tif-IA*), and *TATA-box binding protein-associated factor RNA polymerase I subunit B* (*taf1b*) (Engel et al., 2017; Yuan et al., 2002) were significantly upregulated in *bms1l^sq163/sq163^* at 5dpf (Figure 1B).

Abnormally elevated 45S pre-rRNA production could alter nucleolar morphology (Wang et al., 2016). Immunostaining of Fibrillarin (a nucleolar marker) revealed that, in WT embryos at 5dpf, only ∼8% of hepatocytes contained three or more nucleoli, ∼29% two nucleoli, and ∼63% single nucleolus per hepatocyte (Figure 1C and D). In contrast, in *bms1l^sq163/sq163^*, ∼35% of hepatocytes harbored three or more nucleoli and only ∼33% single nucleolus per hepatocyte (Figure 1C and D). Pancreatic cells behaved similarly (Figure 1E and F). Interestingly, instead of nucleolus number, the area ratio of nucleolus vs. nucleus was increased in *bms1l^zju1/zju^*^*1*^ hepatocytes (10.3%) compared to WT (4.5%) at 5dpf (Figure 1G and H). Consistently, Fibrillarin levels were markedly elevated in both mutants (Figure 1I). Together, these data demonstrate the importance of Bms1l in regulating 18S rRNA maturation and nucleolus architecture.

### Loss-of-function of Bms1l arrests cells at the S-phase but with continuous genomic DNA over-replication

**Figure 2:**
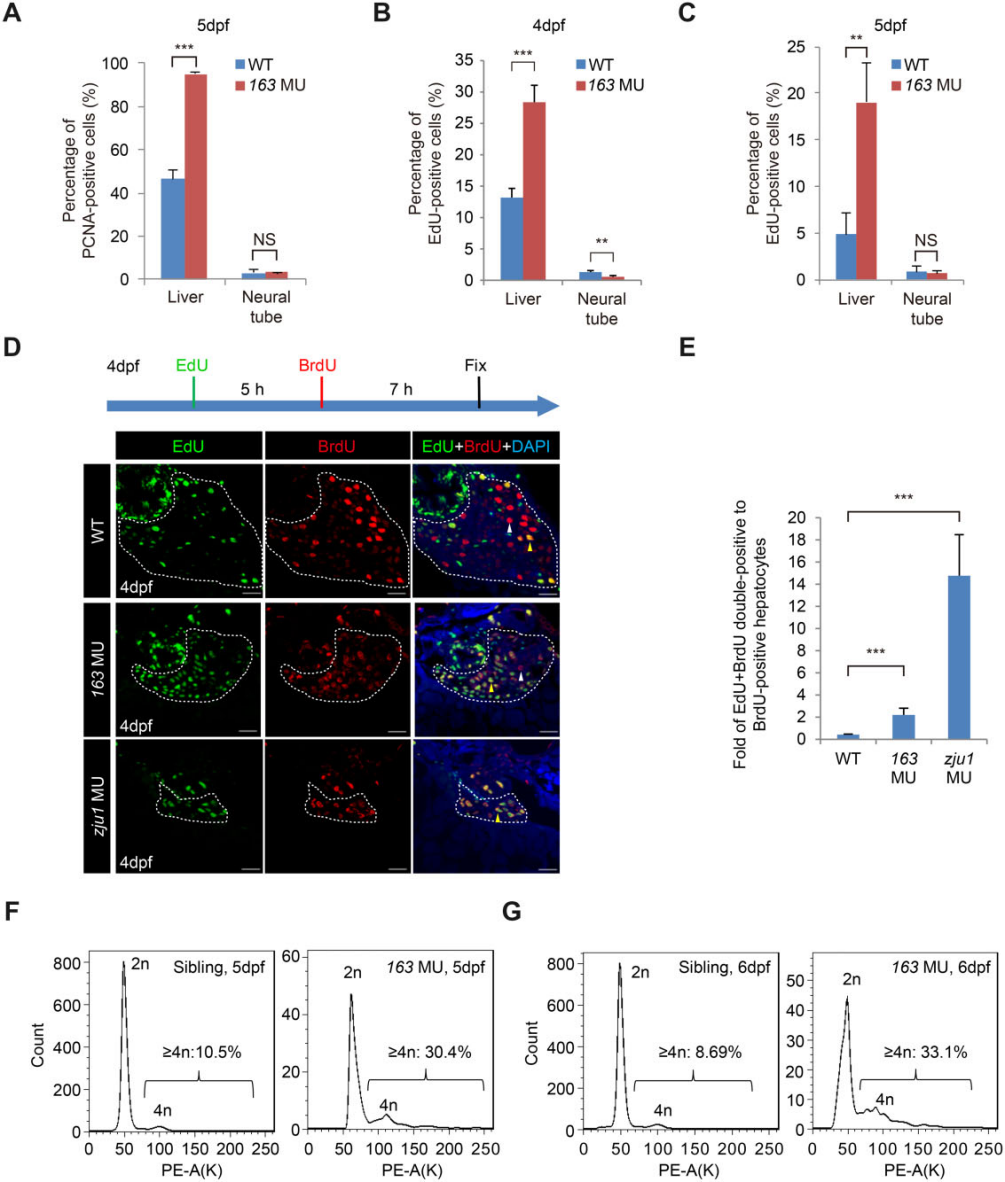
Bms1l mutations cause genomic DNA partial over-replication and arrest cells at the S-phase. (**A**) Immunostaining of PCNA showing the significant increase in the percentage of PCNA-positive cells in *bms1l^sq163/sq163^* hepatocytes but not in the NT when compared with WT at 5dpf. Hepatocyte samples: three WT embryos, 3090 cells counted; three *bms1l^sq163/sq163^* embryos, 1336 cells counted. For NT cells, the pixels of PCNA-positive and DAPI-positive signals were captured by Photoshop and were used to obtain the percentage of PCNA-positive cells as shown. (**B** and **C**) The percentage of EdU-positive hepatocytes were significantly higher in *bms1l^sq163/sq163^* than in WT both at 4dpf (**B**) and 5dpf (**C**). Hepatocyte samples in **B**: four WT embryos, 9422 cells counted; three *bms1l^sq163/sq163^* embryos, 1958 cells counted. In **C**: four WT embryos, 7995 cells counted; three *bms1l^163/sq163^* embryos, 1684 cells counted. (**D **and** E**) EdU and BrdU were sequentially injected into 4dpf embryos as shown (**D**, top panel). Staining of EdU and BrdU was performed (**D**, lower panels). The ratios of BrdU + EdU double-positive vs. BrdU-only-positive hepatocytes were significantly higher in *bms1l^sq163/sq163^* and *bms1l^zju1sq163^* mutants than in WT at 4dpf (**E**), demonstrating DNA over-replication in the two mutants. Samples: five WT embryos, 2591 cells counted; seven *bms1l^163/sq163^* embryos, 1245 cells counted; four *bms1l^zju1/zju1^* embryos, 593 cells counted. Scale bar, 20 μm. (**F** and **G**) Flow cytometry analysis showing a significant increase in the ratio of ≥4n hepatocytes in *bms1l^sq163/sq163^* compared with its siblings (the pool of *bms1^+/+^* and *bms1^sq163/+^*) at 5dpf (**F**) and 6dpf (**G**). Average percentages of ≥4n hepatocytes from three repeats for each genotype were provided. PE-A(K), signal intensity in PE channel. In **A**‒**C**, **E**, and **F**, **P < 0.01, ***P < 0.001; NS, no significance.

The small liver in *bms1l^sq163/sq163^* was attributed to cell cycle arrest (Wang et al., 2012). Surprisingly, immunostaining showed a much higher ratio of PCNA-positive (an S-phase marker) hepatocytes in *bms1l^sq163/sq163^* (94.9%) than in WT (46.4%), while without significant difference in the neural tube (NT) (3.1% in WT vs. 3.2% in *bms1l^sq163/sq163^*) at 5dpf (Figure 2A;  Supplementary Figure S3A and B). An EdU-incorporation experiment (for checking the activity of DNA biosynthesis, so does BrdU labeling) showed that the ratio of EdU-positive hepatocytes was strikingly higher in *bms1l^sq163/sq163^* at both 4dpf (28.4% in *bms1l^sq163/sq163^* vs. 13.2% in WT) (Figure 2B; Supplementary Figure S3C) and 5dpf (19.0% in *bms1l^sq163/sq163^* vs. 4.8% in WT) (Figure 2C). Interestingly, while there was a significant difference in the NT at 4dpf (0.6% in *bms1l^sq163/sq163^* vs. 1.3% in WT) (Figure 2B), no significant difference (0.7% in *bms1l^sq163/sq163^* vs. 0.8% in WT) was observed at 5dpf between WT and *bms1l^sq163/sq163^* (Figure 2C), which coincides with the dynamic expression patterns of *bms1l* during these developmental stages (Wang et al., 2012). We previously showed that *bms1l* transcripts are maternally deposited, which is expected to support early embryogenesis such as NT formation. The zygotic expression of *bms1l* was clearly enriched in the digestive organs but not in the NT at 2.5dpf and 4dpf (Wang et al., 2012), suggesting that the early development of digestive organs but not of the NT likely relies on the zygotic Bms1l. Therefore, Bms1l depletion is expected to have little effect on the NT development at 5dpf and the status of the cell proliferation in the NT can be used as the control to demonstrate that the cell proliferation abnormality in the mutant digestive organs is due to the loss-of-function of *bms1l* rather than a developmental delay.

Data above suggest that the *bms1l^sq163/sq163^* hepatocytes are undergoing active DNA biosynthesis despite of cell cycle arrest. To confirm this, we performed an EdU/BrdU double-labeling pulse-chase experiment. Considering that the retention time of free EdU in the liver is <6 h (Supplementary Figure S3D), we first injected EdU into the embryos at 4dpf and followed by BrdU injection 5 h later. Doubly injected embryos were fixed at 7 h after BrdU injection for staining of EdU and BrdU, respectively (Figure 2D, drawing on the top). The scenario is that if a cell is labeled by EdU only (EdU-positive), this cell has been experiencing the S-phase after EdU injection but completes the S-phase before BrdU injection. On the other hand, if a cell is labeled by BrdU only (BrdU-positive), this cell enters the S-phase only after BrdU injection. For a cell labeled by both EdU and BrdU (EdU + BrdU double-positive), there might be three possibilities: (i) an EdU-positive cell re-enters cell cycle, which can be labeled by BrdU; (ii) an EdU-positive cell has yet completed its S-phase when BrdU is injected; (iii) an EdU-positive cell is arrested at the S-phase but with continuing DNA biosynthesis. Therefore, we anticipated that by comparing the number of BrdU + EdU double-positive cells vs. BrdU-only-positive cells, we could determine the status of DNA biosynthesis in each cell during this period (Figure 2D). Statistics showed that the fold of BrdU + EdU double-labeled to BrdU-only-labeled cells was ∼2.2 and ∼14.8 in the *bms1l^sq163/sq163^* and *bms1l^zju1/zju^*^*1*^ livers, respectively, drastically and significantly higher than that in the WT (0.42) (Figure 2E). These data suggest that mutant hepatocytes are arrested at the S-phase characterized by continuous genomic DNA biosynthesis.

To determine whether continuous genomic DNA biosynthesis finally yields cells containing abnormal DNA contents, we microdissected liver buds from 5dpf and 6dpf WT and *bms1l^sq163/sq163^* embryos in the *Tg(fabp10a: RFP)* genetic background where hepatocytes were specifically labeled by RFP (Her et al., 2003). Liver buds were disassociated and ∼86% of the dissociated cells were confirmed to be hepatocytes by costaining of RFP and the hepatocyte marker Bhmt (Yang et al., 2011; Gao et al., 2018; Supplementary Figure S3E). DNA content analysis of RFP-positive cells by flow cytometry (Guan et al., 2016) showed that the ratio of cells with DNA content ≥4n against total cells were ∼30.4% and ∼33.1% in 5dpf and 6dpf *bms1l^sq163/sq163^* mutants, respectively, much higher than that in the siblings (the pool of *bms1^+/+^* and *bms1^sq163/+^*, 10.5% and 8.7% at 5dpf and 6dpf, respectively) (Figure 2F and G). Notably, the DNA content >4n in the mutant cells did not show distinct peaks, suggesting that these cells suffered from genomic DNA partial over-replication but not polyploidic.

### Loss-of-function of Bms1l upregulates the transcriptome associated with the nucleolar activities

Next, we performed an RNA sequencing (RNA-seq) experiment to determine the effect of Bms1l depletion on the gene expression profiles in 3dpf-old embryos. The qualities of the RNA-seq data from three WT and three *bms1l^zju^*^*1/zju1*^ mutant samples were evaluated by the number of the clean reads (between 5.4G and 6.4G), mapping rates (>84% for all six samples) of the clean sequences to the zebrafish genome ((Danio_rerio.GRCz10.84 from ENSEMBL) and hierarchical clustering analysis (Supplementary Figure S4A and B). Cross comparison of gene expression using the DEseq method based on the reads per kilobase million mapped reads method (Wang et al., 2010; Wagner et al., 2012) identified 453 downregulated genes (log_2_ ≤ −1, *P* < 0.05) and 390 upregulated genes (log_2_ ≥ 1, *P* < 0.05) in the *bms1l^zju^*^*1/zju1*^ mutant embryos (Supplementary Figure 4C, Tables S1 and S2).

**Figure 3:**
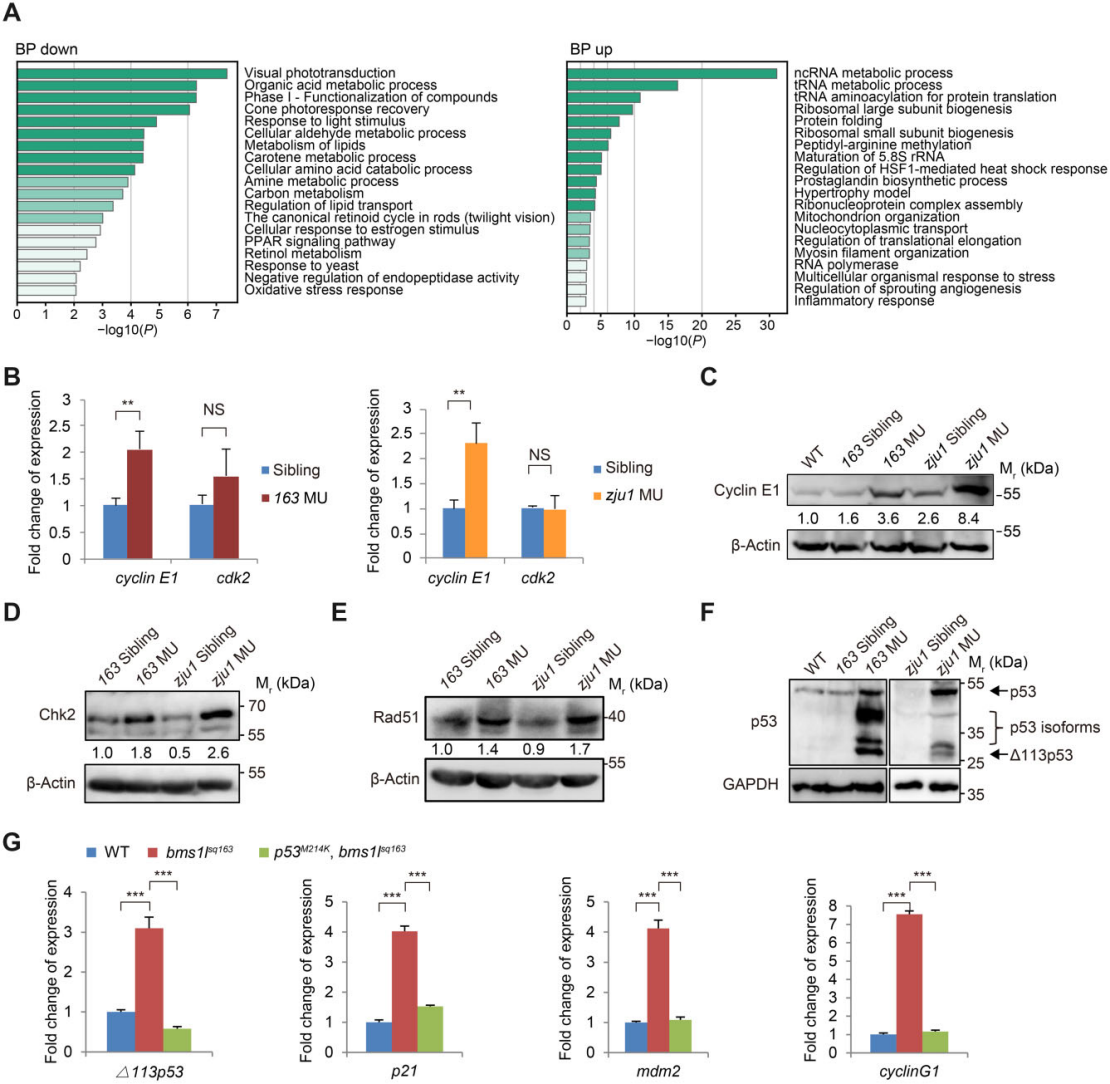
Bms1l mutations activate DNA-damage response. (**A**) GO enrichment analysis of the biological process (BP) category of RNA-seq data from WT and *bms1l^zju1/zju1^* mutant embryos at 3dpf. BP up, enrichment of upregulated genes; BP down, enrichment of downregulated genes. (**B**) qPCR analysis showing the elevated expression of *cyclin E1* but not *cdk2* in both *bms1l^sq163/sq163^* and *bms1l^zju1/zju1^* mutants at 5dpf. (**C**–**F**) Western blotting showing that the protein levels of Cyclin E1 (**C**) and DNA-damage response markers Chk2 (**D**), Rad51 (**E**), p53 and p53 isoforms (**F**) were obviously increased in both *bms1l^sq163/sq163^* and *bms1l^zju1/zju1^* compared with their siblings or WT at 5dpf. β-Actin and GAPDH: loading control. (**G**) qPCR analysis of the expression of △*113p53*, *p21*, *mdm2*, and *cyclinG1* in 5dpf-old *bms1l^sq163/sq163^* mutant, *bms1l^sq163/sq163^p53^M214K/M214K^* double-mutant and WT. The upregulated expression of these four genes in the *bms1l^sq163/sq163^* mutant was restored to the WT level in *bms1l^sq163/sq163^p53^M214K/M214K^* double-mutant. *163* MU, *bms1l^sq163/sq163^*; *zju1* MU, *bms1l^zju1/zju1^*. In **B** and **G**, **P < 0.01, ***P < 0.001; NS, no significance.

We then performed a gene ontology (GO) (Ashburner et al., 2000) analysis of the 453 downregulated genes in *bms1l^zju^*^*1/zju1*^ (Supplementary Table S1). The top 20 significantly affected items in the GO biological process (GO_BP) category were related to photoresponse and metabolic activities (Figure 3A, left panel), including genes involved in response to light stimulus (9 genes), organic acid metabolic process (27 genes), metabolism of lipids (17 genes), etc. (Supplementary Table S3), which coincides with the hypoplastic phenotype of the digestive organs exhibited by *bms1l^zju^*^*1/zju1*^. For the 390 upregulated genes in *bms1l^zju^*^*1/zju1*^, the top 20 significantly affected items in the GO_BP category were mainly related to the ribosome biogenesis and tRNA processing (Figure 3A, right column), including genes involved in ncRNA metabolic process (49 genes), tRNA metabolic process (24 genes), ribosomal large subunit biogenesis (12 genes), ribosomal SSU biogenesis (9 genes), etc. (Supplementary Table S4), which is consistent with the upregulation of rRNA expression (Figure 1A and B) and increase of the nucleolar volume observed in *bms1l^zju^*^*1/zju1*^ (Figure 1A‒H). Furthermore, we noticed that genes related to the cell cycle regulation, including *cyclin E1*, *malignant T cell amplified sequence 1*, and *NIMA-related kinase 8* were significantly upregulated in *bms1l^zju^*^*1/zju1*^ at 3dpf (Supplementary Figure 4D), reflecting the abnormal cell cycle progression and genomic DNA replication observed in *bms1l^zju^*^*1/zju1*^ (Figure 2A‒G).

### Loss-of-function of Bms1l activates DNA-damage response

Consistent with the RNA-seq data (Supplementary Figure 4D), a qPCR analysis showed that the expression level of *cyclin E1* but not *CDK2* was significantly higher in *bms1l^sq163/sq163^* and *bms1l^zju1/zju^*^*1*^ with respect to their siblings (Figure 3B). Consequently, the level of Cyclin E1 protein was elevated in both mutants (Figure 3C). Cyclin-dependent kinase 2 (CDK2) is essential for the G1/S-phase transition during cell cycle. Cyclin E1 is a member of the highly conserved cyclin family, which regulates the function of CDK2 by forming a complex with CDK2. The timing expression of Cyclin E1 plays a direct role in the initiation of DNA replication (Keaton, 2007; Malumbres, 2014; Saldivar et al., 2018). The upregulation of Cyclin E1 nicely coincides with the genomic DNA partial over-replication in *bms1l^sq163/sq163^* and *bms1l^zju1/zju^*^*1*^ hepatocytes (Berger et al., 2005).

Genomic DNA partial over-replication is expected to cause DNA-damage response (Alexander and Orr-Weaver, 2016). Indeed, protein levels of Chk2 and Rad51, two DNA-damage response factors, were highly elevated in both mutants at 5dpf (Figure 3D and E). We also observed an obvious upregulation of p53 in both mutants (Figure 3F), together with upregulation of p53-responsive genes *Δ113p53*, *p21*, *mdm2*, and *cyclin G1* (Berghmans et al., 2005; Chen et al., 2005, 2009; Supplementary Figure S5A) and the Δ113p53 family proteins (Figure 3F; Shi et al., 2015). Strikingly, the increased p53 and Δ113p53 were highly enriched in the nucleolus of the mutant hepatocytes (Supplementary Figure S5B). Introducing the *p53^M214K^* mutation (Berghmans et al., 2005) to the *bms1l^sq163^* mutant (i.e. *p53^M214K/M214K^bms1l^sq163/sq163^* double-homozygous mutant) reduced the transcripts of the p53 target genes *Δ113p53*, *p21*, *mdm2*, and *cyclin G1* (Figure 3G). Interestingly, the sizes of liver and pancreas were only partially recovered (Supplementary Figure S5C‒F), suggesting that Bms1l is a multifunctional protein.

### Loss-of-function of Bms1l causes replication-fork stall

**Figure 4:**
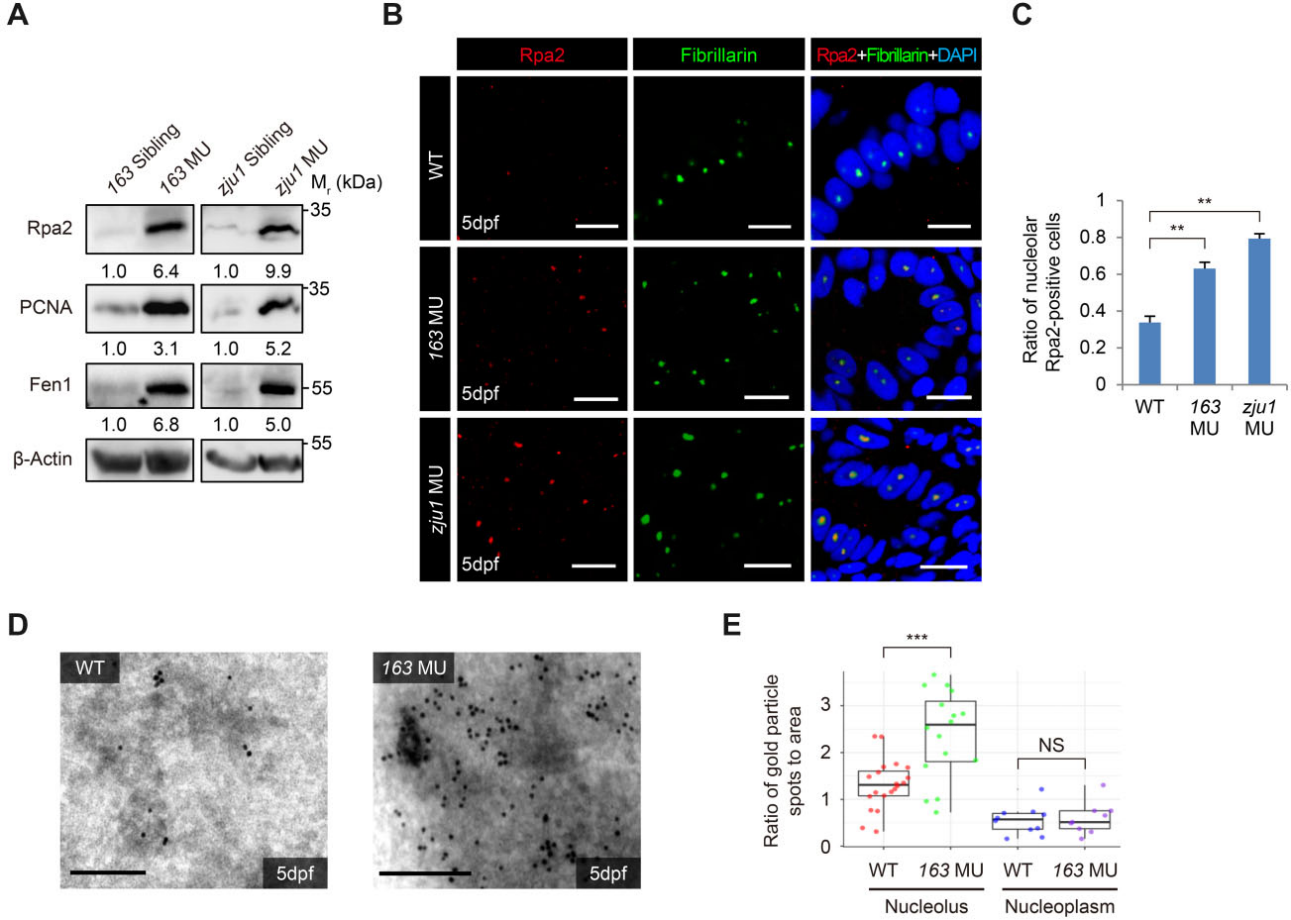
Bms1l mutations cause replication fork stall. (**A**) Western blotting showing a drastic increase of Rpa2, PCNA, and Fen1 protein levels in 5dpf *bms1l^sq163/sq163^* and *bms1l^zju1/zju1^* mutants compared with their siblings. β-Actin: loading control. (**B** and **C**) Coimmunostaining of Rpa2 and Fibrillarin in gut epithelia was performed (**B**). The ratio of the nucleolar Rpa2-positive cells was significantly higher in *bms1l^sq163/sq163^* and *bms1l^zju1/zju1^* mutants than in WT at 5dpf (**C**). Samples: two WT embryos, 544 cells examined; three *bms1l^163/sq163^* embryos, 845 cells examined; two *bms1l^zju1/zju1^* embryos, 277 cells examined. Scale bar, 10 μm. (**D** and **E**) Representative images showing Immuno-TEM of Rpa2 in hepatocytes dissected from the WT and *bms1l^sq163/sq163^* embryos at 5dpf (**D**). Statistical analysis showing a significant accumulation of Rpa2-positive gold particles in the nucleoli but not in the nucleoplasm in *bms1l^sq163/sq163^* hepatocytes compared with WT (**E**). For each genotype, 17‒20 nucleoli were examined. Each dot represents the ratio of gold particle numbers against area of the nucleolus or nucleoplasm in one cell. Scale bar, 0.2 μm. In **C** and **E**, **P < 0.01, ***P < 0.001; NS, no significance.

Next, we examined PCNA, Rpa2, and Fen1, hallmarks for the DNA replication fork progression (Shen et al., 2005) and found that all were highly upregulated in *bms1l^sq163/sq163^* and *bms1l^zju1/zju^*^*1*^ at 5dpf (Figure 4A). Strikingly, immunostaining analysis revealed the accumulation of Rpa2 in the nucleoli (Figure 4B) and the ratio of nucleolar Rpa2-positive cells was significantly increased in both mutants (Figure 4C). Immuno-transmission electron microscopy (immuno-TEM) analysis of liver cells from 5dpf WT and *bms1l^sq163/sq163^* embryos in the *Tg(fabp10a: RFP)* genetic background identified a large number of Rpa2-positive gold particles localized in the nucleolus in *bms1l^sq163/sq163^*, in contrast to only a few in WT (Figure 4D;  Supplementary Figure S6). Compared with a significant increase of Rpa2-positive gold particles in the mutant nucleoli, no significant difference was observed in the nucleoplasm between WT and *bms1l^sq163/sq163^* hepatocytes (Figure 4E). Therefore, replication fork is stalled in *bms1l^sq163/sq163^*, prominently in the nucleoli.

### Ttf1 is accumulated in the nucleolus in bms1l mutants

**Figure 5:**
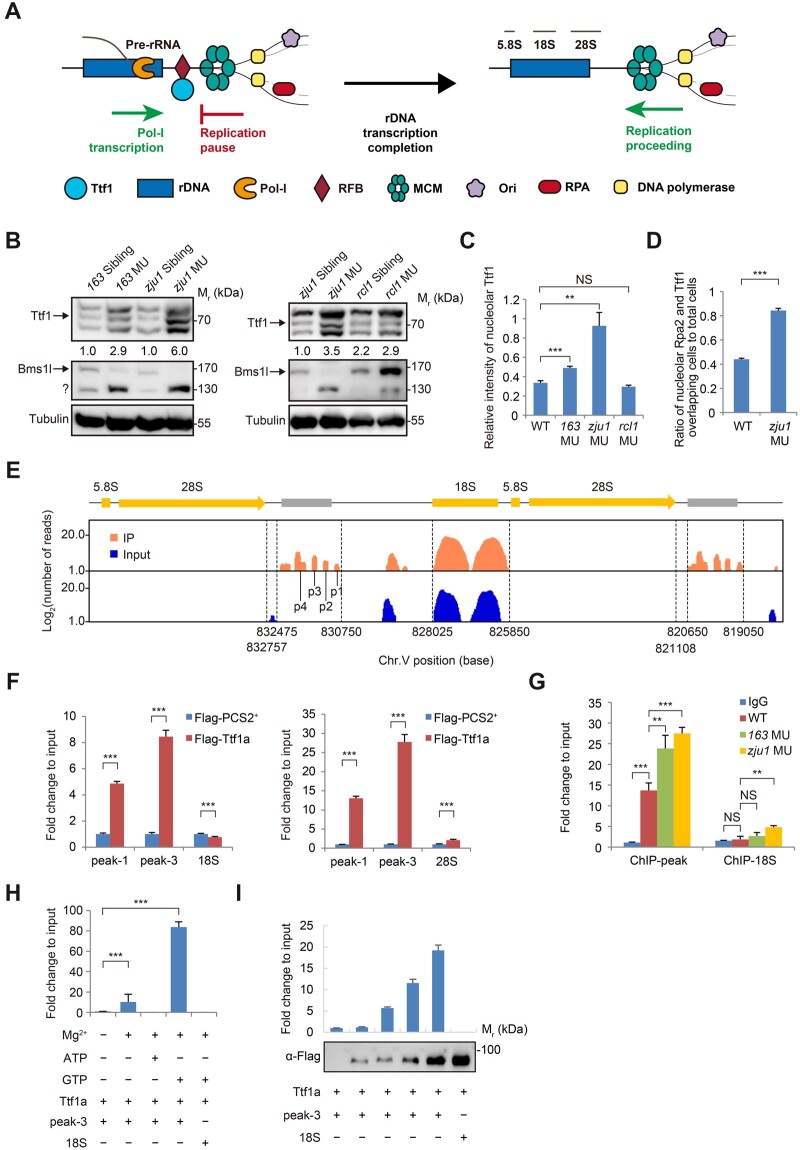
Ttf1 is accumulated on its binding sites in *bms1l* mutants. (**A**) A drawing to illustrate the head-on conflict between rDNA transcription and replication during the S-phase in higher eukaryotes. Binding of Ttf1 to the RFB-site prevents the conflict. Once the transcription is completed, Ttf1 is proposed to leave the RFB-site to allow progression of the replication fork; however, how Ttf1 is dissociated from the RFB-site remains unknown. (**B**) Western blotting showing an increase of Ttf1 protein level in *bms1l^sq163/sq163^* and *bms1l^zju1/zju1^* but not in *rcl1^−/−^* compared with their corresponding siblings at 5dpf. Tubulin: loading control. ?: unknown protein. (**C**) Signal intensity of nucleolar Ttf1 in the gut epithelia was significantly higher in *bms1l^sq163/sq163^* and *bms1l^zju1/zju1^* than in WT and *rcl1^−/−^*. Samples: three WT embryos, 364 cells counted; three *bms1l^sq163/sq163^* embryos, 183 cells counted; three *bms1l^zju1/zju1^* embryos, 98 cells counted; three *rcl1^−/−^* embryos, 176 cells counted. (**D**) Ratio of the nucleoli displaying intervening signals of Ttf1 and Rpa2 in the gut epithelia was significantly higher in *bms1l^zju1/zju1^* than in WT. Samples: three WT embryos, 264 cells examined; three *bms1l^zju1/zju1^* embryos, 140 cells examined. (**E**) ChIP‒seq identification of Ttf1-binding sites (peak-1 to peak-4) downstream of the 3ʹ-end of rDNA gene on chromosome 5 (Chr.V). (**F**) ChIP‒qPCR analysis showing the strong binding of Flag-tagged Ttf1a to peak-1 and peak-3 but not to the 18S (left panel) or 28S (right panel) rDNA. (**G**) Endogenous Ttf1 showed significant enrichment on its binding sites (ChIP-peak: peak-1 to peak-4) in *bms1l^sq163/sq163^* (*163* MU) and *bms1l^zju1/zju1^* (*zju1* MU) than in WT at 3dpf. IgG and ChIP-18S: negative controls. (**H** and **I**) ChIP‒qPCR analysis showing that, in the presence of Mg^2+^, the binding of purified Ttf1a to peak-3 depends on GTP but not ATP (**H**) and is correlated to the dosage of purified Ttf1a (**I**). Lower panel in **I**: Ttf1a was examined by western blotting. In **C**, **D**, and **F**‒**H**, **P < 0.01, ***P < 0.001; NS, no significance.

The head-on conflict between rDNA transcription and replication during the S-phase in eukaryotes is resolved by the RFBs and the associating factors such as TTF1 in human and mouse (Figure 5A; Gerber et al., 1997; Kobayashi, 2003; Mirkin and Mirkin, 2007; Akamatsu and Kobayashi, 2015; Hamperl and Cimprich, 2016). The upregulation of rDNA transcription and replication fork stall in *bms1l^sq163/sq163^* and *bms1l^zju1/zju^*^*1*^ prompted us to explore whether Bms1l plays a role in facilitating replication-fork progression at the rDNA loci. The Myb-like DNA-binding protein Ttf1 is a key head-on RFB-site-binding factor at the rDNA loci (Bartsch et al., 1987; Gerber et al., 1997; Akamatsu and Kobayashi, 2015). Zebrafish genome contains two closely linked homologous *ttf1* genes on chromosome 5, namely *ttf1a* and *ttf1b* (Supplementary Figure S7A‒C). Sequencing analysis revealed that except for 2dpf (∼40%), over 60% of the transcripts represented *ttf1a* from 3dpf to 5dpf (Supplementary Figure S7D). The identity of the endogenous Ttf1 was determined based on an antibody detecting both Ttf1a and Ttf1b proteins (Supplementary Figure S7E) and a MO targeting the translation start codon (TSC) ATG region (identical in *ttf1a* and *ttf1b*) (Supplementary Figure S7F and G) for knockdown of the endogenous Ttf1a/Ttf1b protein expression (Supplementary Figure S7H). Total Ttf1, as Bms1l, was expressed in embryos from 2dpf to 5dpf (Supplementary Figure S7I).

The complex formed by Bms1 and Rcl1 is essential for ribosome biogenesis (Billy et al., 2000; Wegierski et al., 2001; Delprato et al., 2014; Kornprobst et al., 2016; Wang et al., 2016; Cheng et al., 2017; Zhu et al., 2021). The zebrafish genome contains a single copy of the *rcl1* gene and *rcl1*-null mutant (*rcl1*^*−/−*^) dies before 15dpf (Wang et al., 2016; Zhu et al., 2021). Interestingly, Ttf1 level was obviously elevated in both *bms1l^sq163/sq163^* and *bms1l^zju1/zju^*^*1*^ mutants (Figure 5B, left panels) but not in *rcl1*^*−/−*^ mutant at 5dpf (Figure 5B, right panels). In addition, neither Rpa2 nor Fen1 was elevated in *rcl1*^*−/−*^ (Supplementary Figure S8A). Immunostaining showed that Ttf1 in *bms1l^sq163/sq163^* and *bms1l^zju1/zju^*^*1*^, but not in *rcl1*^*−/−*^, was accumulated in the nucleoli of the gut epithelia (Figure 5C;  Supplementary Figure S8B). Importantly, coimmunostaining analysis showed that ∼84% of the *bms1l^zju1/zju^*^*1*^ gut epithelia cells exhibited intervening and overlapped staining of Rpa2 and Ttf1 in the nucleoli, while only ∼44% was observed in WT (Figure 5D;  Supplementary Figure S8C). These data suggest that the observed replication fork stall at the rDNA loci in *bms1l^zju1/zju^*^*1*^ is likely due to accumulation of Ttf1 on the head-on RFB-sites but is independent of Rcl1.

### ChIP‒seq identification of zebrafish Ttf1-binding sites (RFB-sites)

Zebrafish genome contains two main rDNA loci, the maternal type (M-type rDNA, located on chromosome 4, whose transcripts were detected in the egg and female gonad) and the somatic type (S-type, located on chromosome 5, transcribed in embryos and different tissues/organs) (Locati et al., 2017; Tao et al., 2020). A chromatin immunoprecipitation sequencing (ChIP‒seq) analysis (GEO accession number: GSE176455) revealed that Ttf1 bound to DNA sequences in 35 regions in the zebrafish genome (Supplementary Tables S5 and S6), including four homologous regions, namely peak-1 to peak-4, downstream of the somatic rDNA gene on chromosome 5 at 3dpf (Figure 5E;  Supplementary Figure S9A). Notably, the sequences in these four peaks do not contain an obvious consensus DNA sequence (AGGTCGACCAGATTANTCCG) for human Ttf1 (Supplementary Figure S9A; Bartsch et al., 1987, 1988), which might owe to the low identity (30%) between their Myb-like DNA-binding domains in zebrafish Ttf1a and human TTF1.

We then expressed FLAG-tagged Ttf1a (cloned into the *pCS2^+^* expression vector) in 293 T cells and extracted the protein for incubation with synthesized peak-1 and peak-3 sequences (using the protein from the *pCS2^+^* vector-transfected cells as the control). ChIP‒qPCR analysis showed that Ttf1a strongly bound to peak-1 and peak-3 sequences (Figure 5F;  Supplementary S9B). Furthermore, a ChIP‒PCR analysis revealed that the occupancy of the endogenous Ttf1 on its binding sites was significantly enriched in *bms1l^sq163/sq163^* and *bms1l^zju1/zju^*^*1*^ when compared with that in WT at 3dpf (Figure 5G). Finally, we purified Flag-tagged Ttf1a expressed in 293 T cells (Supplementary Figure S9C). Upon mixing, the binding of the purified Ttf1a to the peak-3 sequence was depended on the presence of GTP but not ATP (Figure 5H) in a dosage-dependent manner (Figure 5I). Therefore, zebrafish Ttf1 binds to specific DNA sites within the rDNA locus.

### Bms1l interacts with Ttf1 and directly dissociates the Ttf1‒RFB complex with its GTPase activity

**Figure 6:**
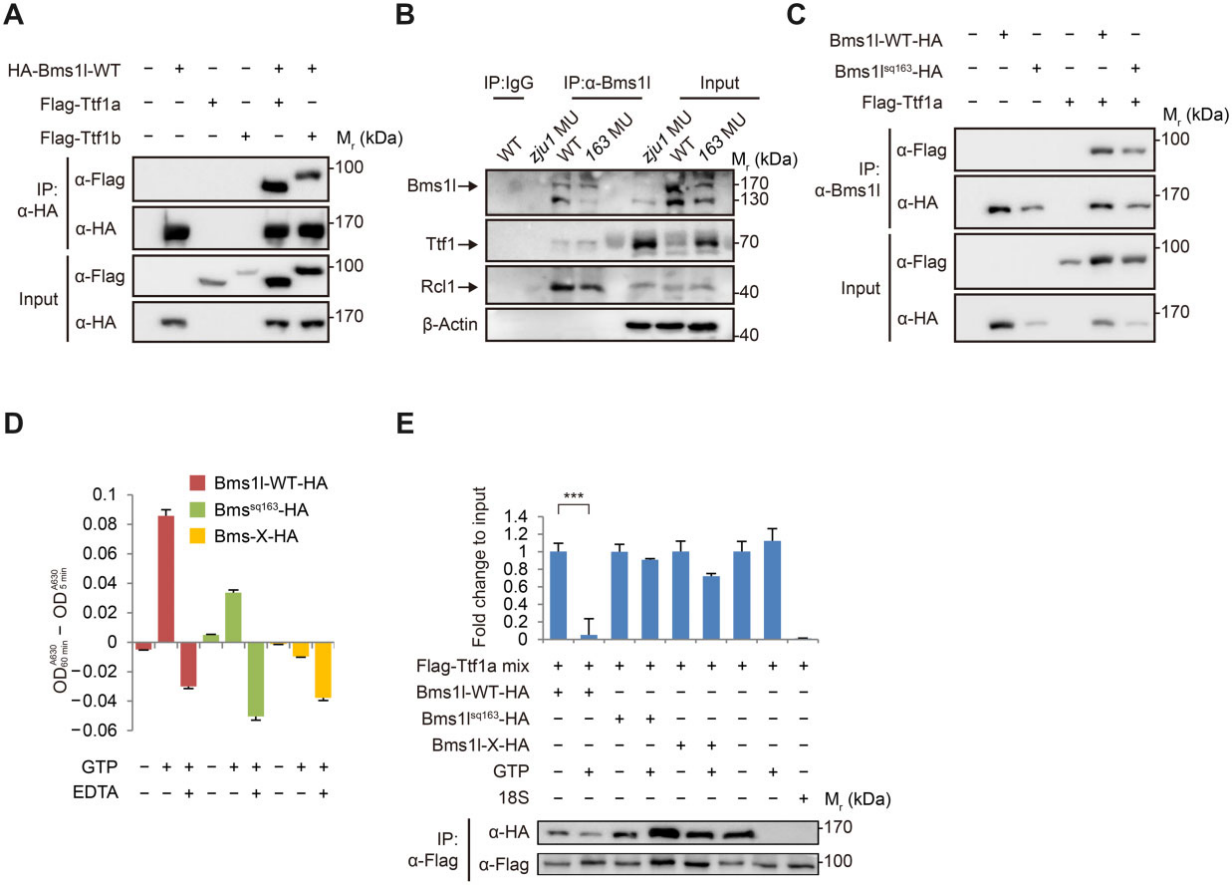
Bms1l interacts with Ttf1 and displaces Ttf1 from DNA-binding site with its GTPase activity. (**A**) Co-IP analysis showing that the overexpressed Bms1l (Bms1l-WT-HA) interacts with both Ttf1a and Ttf1b (Flag-tagged). Total protein was extracted from 293 T cells at 48 h after transfection with corresponding plasmids as shown and was subjected to Co-IP using an HA-tag antibody. (**B**) Co-IP analysis showing the successful pulldown of endogenous Ttf1 and Rcl1 by endogenous Bms1l or Bms1l^163^. β-Actin: loading control. (**C**) Co-IP analysis showing that the overexpressed Ttf1a (Flag-tagged) interacts with both Bms1l and Bms1l^163^ (HA-tagged). Total protein was extracted from 293 T cells at 48 h after transfection with corresponding plasmids as shown and was subjected to Co-IP using an anti-Bms1l antibody. (**D**) GTPase activity assay using purified proteins showing that Bms1l-X-HA is enzymatically inactive, while Bms1l^sq163^-HA exhibits ∼43% of Bms1l-WT-HA activity. Free phosphate concentration in each reaction was obtained by A630 color absorbance at 5 and 60 min, respectively. The subtracted value between 60 and 5 min is presented as the relative GTPase activity. (**E**) ChIP‒qPCR analysis for the effect of Bms1l on dissociation of the Ttf1a‒DNA complex. Purified Ttf1a was mixed with the peak-3 DNA and 10 µM GTP (Flag-Ttf1a mix). The mixture was then incubated with purified Bms1l-WT, Bms1l-163, or Bmsl1-X with or without 50 µM GTP for 60 min. Only Bms1l-WT-HA obviously dissociates the Ttf1a‒DNA complex. Lower panels: the Co-IP products (Ttf1a, Bms1l, or derivatives) were examined by western blotting with respective antibodies. ***P < 0.001.

Coimmunoprecipitation (Co-IP) showed that Bms1l interacted with both Ttf1a and Ttf1b (Figure 6A). Co-IP analysis of proteins extracted from 3dpf WT embryos showed that endogenous Ttf1, albeit less than Rcl1, was successfully pulled down by Bms1l (Figure 6B;  Supplementary Figure S10A). Interestingly, both endogenous and overexpressed Bms1l^sq163^ mutant proteins could interact with Ttf1 (Figure 6B and C).

We purified HA-tagged WT Bms1l, Bms1l^sq163^, and Bms1l-X (a GTPase-inactivated mutant, Supplementary Figure S10B; Leipe et al., 2002) proteins expressed in 293 T cells via affinity column (Supplementary Figure S10C). GTPase activity assay showed that the GTPase activity of the Bms1l^sq163^ mutant protein was greatly compromised, while the Bms1l-X mutant protein displayed negligible GTPase activity (Figure 6D;  Supplementary Figure S10D).

To determine whether Bms1l could dissociate the Ttf1‒DNA complex, we first mixed the purified Ttf1a with the peak-3 DNA and GTP (10 µM). This mixture was then incubated with purified HA-tagged Bms1l-WT, Bms1l^sq163^, and Bms1l-X proteins with or without 50 µM GTP (Figure 6E;  Supplementary Figure S10E), respectively. We found that although all three proteins were able to interact with Ttf1, only Bms1l-WT robustly displaced Ttf1a from the peak-3 DNA (Figure 6E). Since Bms1l-X is a GTPase-inactivated mutant (Leipe et al., 2002) and Bms1l^sq163^ exhibited only 43% of the WT Bms1l activity (Figure 6D), we concluded that dissociation of the Ttf1‒DNA complex apparently depends on the Bms1l GTPase activity.

### Knockdown of human BMS1 causes DNA over-replication and nucleolar accumulation of RPA2

**Figure 7:**
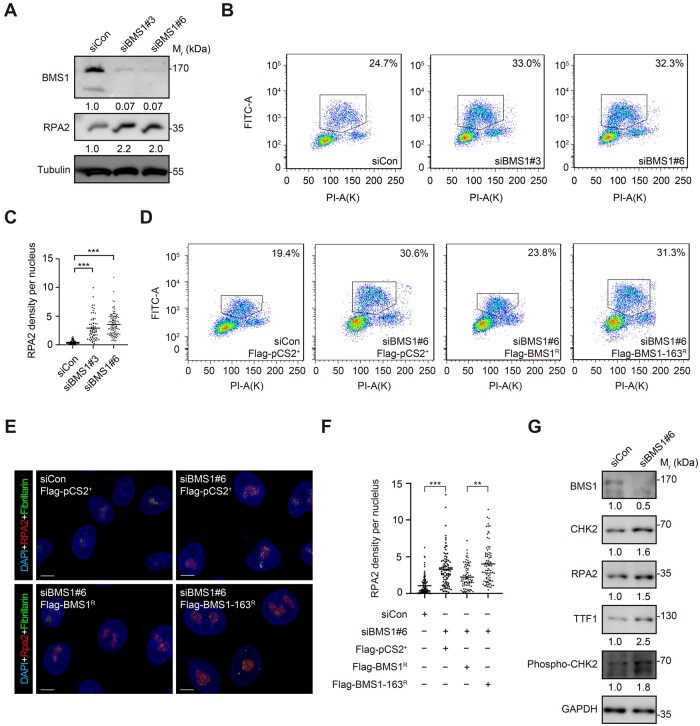
Knockdown of human BMS1 causes DNA over-replication and nucleolar accumulation of RPA2. (**A**) Western blotting of human BMS1 and RPA2 in HeLa cells showing that knockdown of BMS1 by siBMS1#3 and siBMS1#6 treatment obviously increases the level of RPA2 compared with siControl (siCon) treatment at 96 h. Tubulin: loading control. (**B**) Flow cytometry analysis of BrdU/PI double-labeled cells at 96 h after siRNA transfection showing that knockdown of human BMS1 arrests cells at the S-phase. Frame: ratio of cells at S-phase; PI-A, PI intensity; FITC-A, BrdU intensity. (**C**) Statistical analysis showing a nucleolar enrichment of RPA2 after siBMS1#3 and siBMS1#6 treatment. (**D**) Flow cytometry analysis of BrdU/PI double-labeled cells showing the rescue of the ratio of cells at the S-phase by Flag-BMS1^R^ but not Flag-BMS1-163^R^ in siBMS1#6-treated cells when compared with Flag-pCS2^+^. Frame: ratio of cells at S-phase. (**E** and **F**) Coimmunostaining of RPA2 and Fibrillarin (**E**) showing that the nucleolar enrichment of RPA2 after siBMS1#6 treatment is alleviated by Flag-BMS1^R^ but not Flag-BMS1-163^R^ (**F**). Scale bar, 5 μm. (**G**) Western blotting showing the upregulation of CHK2, phosph-CHK2 (T68), and TTF1 in Hela cells after BMS1 knockdown. In **C** and **F**, **P < 0.01, ***P < 0.001.

Two siRNAs (siBMS1#3 and siBMS1#6) specifically targeting human *BMS1* were designed and used to knockdown BMS1 protein expression in Hela cells (Figure 7A;  Supplementary Table S7). BrdU/propidium iodide (PI) double-labeling showed that knockdown of BMS1 increased the ratio of S-phase cells (siControl: 24.7%; siBMS1#3: 33.0%; siBMS1#6: 32.3%) (Figure 7B), together with an elevation of RPA2 protein level (Figure 7A) and significant enrichment of RPA2 in the nucleoli in BMS1-knockdown cells (Figure 7C;  Supplementary Figure S11A). We then obtained siBMS1#6-resistant WT *bms1* (Flag-BMS1^R^) and mutant *bms1^sq163^* (Flag-BMS1-163^R^) constructs through site-guided mutagenesis but without altering the open-reading frame (Supplementary Table S4). The two plasmids were transfected into Hela cells, which have been transfected with siBMS1#6 24 h earlier (Supplementary Figure S11B). Flow cytometry analysis of BrdU/PI-labeled cells showed that the elevated ratio of S-phase cells by siBMS1#6 (30.6%) were largely restored by Flag-BMS1^R^ (23.8%) when compared with the siControl (19.4%), whilst Flag-BMS1-163^R^ (31.3%) failed to alleviate the effect of siBMS1#6 (Figure 7D), suggesting that, as that observed in the *bms1l^sq163^* zebrafish mutant, human BMS1-163^R^ is also malfunctioning. The enrichment of RPA2 in the nucleolus in the siBMS1#6-treated cells was alleviated by Flag-BMS1^R^ but was visually more enhanced by Flag-BMS1-163^R^ (Figure 7E and F). Furthermore, western blotting results showed that, like RPA2, CHK2, Phospho-CHK2(T68), and TTF1 were obviously upregulated in BMS1-knockdown Hela cells. Therefore, as in zebrafish, knockdown of human BMS1 also causes genomic DNA over-replication and impedes the replication fork progression (Figure 7G).

## Discussion

**Figure 8:**
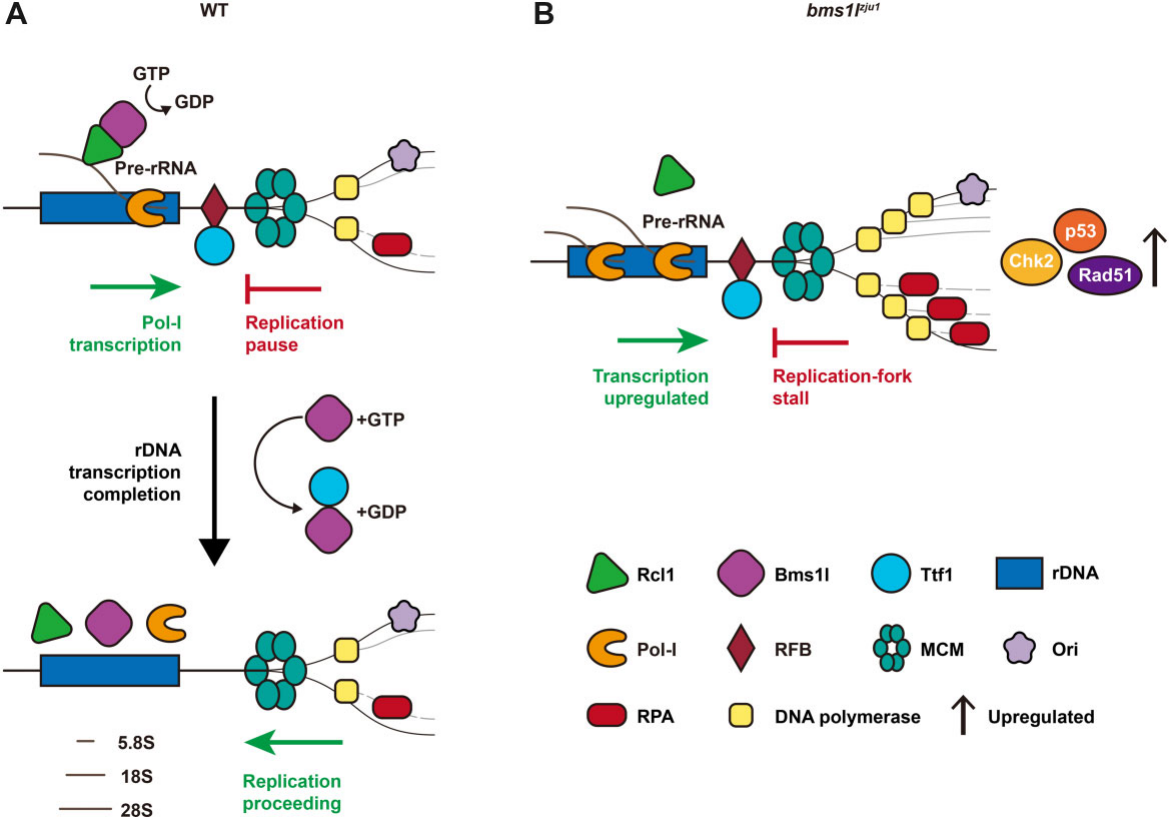
A trio by Bms1l, Rcl1, and Ttf1 resolves the head-on conflict between rDNA transcription and replication. (**A**) A model to propose Bms1l as a mediator in balancing ribosome biogenesis through interaction with Rcl1 and replication fork progression through interaction with Ttf1. (**B**) Compromising Bms1 function not only impairs ribosome biogenesis but also accumulates Ttf1 on RFB-sites to cause replication fork stall together with genomic DNA partial over-replication and cell cycle arrest at the S-phase.

Organogenesis is characterized by cell specification and proliferation. During cell proliferation, DNA replication at the rDNA loci is poised to at least two main challenges: (i) how to ensure completion and accuracy of replication, since pre-rRNA gene is tandemly repeated on the chromosome and (ii) how to resolve the conflict between rDNA transcription and replication, since Pol-I activity is vigorous during the S-phase (Kobayashi, 2003; Mirkin and Mirkin, 2007; Hamperl and Cimprich, 2016). Based on our data, we propose that Bms1 in both zebrafish and human mediates the communication between rDNA transcription and replication with its GTPase activity at the S-phase. In WT, Bms1l interacts with Rcl1 to initiate the pre-rRNA processing. When pre-rRNA processing is near completion (which is coupled with completion of transcription), Bms1 turns to interact with Ttf1 to displace the latter from the head-on RFB-sites to allow the head-on replication fork to proceed (Figure 8A). When Bms1l GTPase activity is compromised, rDNA transcription is upregulated due to the impaired pre-rRNA processing (Figure 8B). Meanwhile, Ttf1 remains on RFB-sites blocking the head-on replication fork progression. Partial over-replication of the genomic DNA triggers DNA-damage response by upregulation of the expression of factors such as Chk2 and Rad51 (Figure 8B). Combination of cellular stresses, including DNA-damage response and accumulation of aberrantly cleaved rRNA transcripts, activate the p53 pathway (Figure 8B). All of these are likely the reasons to cause the cell cycle arrest at the S-phase observed in *bms1l^sq163/sq163^* and *bms1l^zju1/zju^*^*1*^ mutants and in Bms1-koncdown Hela cells. Therefore, nucleolar factors Bms1l and Ttf1 are essential for cell cycle progression at the rDNA loci during the S-phase.

It is surprising that GTP is needed for effective binding of Ttf1 to its target DNA. One possibility is that GTP might help to shift the conformation of Ttf1 to bind to DNA with a high affinity. Resolving the structure of Ttf1 with or without GTP in the future will answer this intriguing question. We observed that, in the absence of Bms1l, mutant cells undergo continuous genomic DNA re-replication even though DNA-damage response and p53 pathway are activated, suggesting that Bms1l might also play a role, directly or indirectly, in the regulation of the G1-to-S transition, which is independent to its duet with Ttf1. It would be interesting in the future to check the status of ataxia‒telangiectasia and Rad3-related, the key regulator of G1-to-S transition (Saldivar et al., 2018), and chromatin licensing and DNA replication factor 1 (Cdt1, a regulator of DNA re-replication) (Roukos et al., 2011) or other key cell cycle regulators in *bms1l^sq163/sq163^* and *bms1l^zju1/zju^*^*1*^ mutants, which might allow us to understand more about the role of Bms1l in the G1/S transition.

Ribosomopathies are named for the category of diseases caused by defective ribosome function or production (Farley-Barnes et al., 2019; Huang et al., 2020). It is worth investigating whether this function of Bms1 is conserved in mammals. A thorough investigation of the full spectrum of Bms1l-interacting proteins will not only allow us to ravel the role of Bms1l in ribosome biogenesis and regulation of cell cycle progression but also shed light on the understanding of ribosomopathies from the views of both defective ribosome production and cell cycle progression.

## Materials and methods

### Zebrafish lines and maintenance

Zebrafish AB line was used as the WT control in this work. Fish were raised and maintained according to standard procedures. *bms1l^sq163^* and *p53^M214K^* mutant lines and transgenic line *Tg(lfabp: RFP; elaA: EGFP)* were obtained as previously described (Berghmans et al., 2005; Wan et al., 2006; Wang et al., 2012). The *bms1l^zju^*^*1*^ mutant line and *rcl1*^*−/−*^ mutant line were generated by CRISPR‒Cas9 technology as detailed in Supplementary Materials and methods. Homozygous mutant embryos (e.g. *bms1l^sq163/sq163^* and *bms1l^zju1/zju^*^*1*^) were obtained by PCR-based genotyping of the progenies derived from the crosses between heterozygous (e.g. *bms1l^sq163/+^* or *bms1l^zju1/+^*) male and female fish. Considering the progenies from one cross were raised in the same container, the pool of the *bms1l^+/+^* (i.e. WT) and heterozygous (i.e. *bms1l^sq163/+^* or *bms1l^zju1/+^*) progenies, being called ‘the sibling’ in this work, was sometimes used as a control.

### Cell lines, plasmids, and siRNA transfection

Human Hela and 293 T cells were used to express different target proteins by plasmid transfection as detailed in Supplementary Materials and methods. Corresponding siRNA sequences for gene knockdown and primer sequences for constructing plasmids are listed in Supplementary Table S7.

### Plasmid construction

Zebrafish WT *bms1l*, *bms1l^sq163^*, *ttf1a*, and *ttf1b* full-length cDNA for constructing *pCS2^+^-HA-Bms1l-WT*, *pCS2^+^-HA-Bms1l-163*, *pCS2^+^-Flag-Ttf1a*, and *pCS2^+^-Flag-Ttf1b* plasmids were obtained through reverse transcription‒PCR (RT‒PCR) using appropriate primer pairs (Supplementary Table S7). *pCS2^+^-Flag-Ttf1-P1*, *pCS2^+^-Flag-Ttf1-P2*, and *pCS2^+^-Flag-Ttf1-P3* sub-clones were derived from the *pCS2^+^-Flag-Ttf1a* plasmid.

### Northern blotting, 18S/28S ratio analysis, and qPCR

Total RNA was extracted from WT, mutant siblings, and mutant embryos using Trizol Reagent (Invitrogen, 15596-026). The digoxigenin (DIG)-labeled 5ʹ-ETS and ITS1 DNA probes were prepared and RNA gel blot hybridization and qPCR was performed as described previously (Chen et al., 2009; Wang et al., 2012). For 18S/28S ratio analysis, total RNA was analyzed by Agilent Bioanalysis 2100 (Agilent). Primer pairs used for qPCR analysis are listed in Supplementary Table S8.

### Whole-mount in situ hybridization

*fabp10a*, *ifabp*, and *trypsin* gene fragments were respectively cloned into the pGEM-T vector to produce DIG-labeled RNA probes (Roche DIG RNA Labeling mix 11277073901) for whole-mount in situ hybridization as previously described (Chen et al., 2005).

### Protein analysis and preparation of antibodies

Total protein extraction from zebrafish embryos or cultured cells, western blotting analysis, and Co-IP assays were performed as previously described (Chen et al., 2009; Guan et al., 2016). All antibodies used in this study are listed in Supplementary Materials and methods. Detailed protocols are described in Supplementary Materials and methods.

### Cryo-sectioning and immunostaining

For cryo-sectioning, embryos were fixed, embedded, and sectioned as detailed in Supplementary Materials and methods. Immunostaining was performed as described (Wang et al., 2016) and also detailed in Supplementary Materials and methods. All immunofluorescence staining images were taken under an Olympus BX61WI confocal microscope.

### EdU and BrdU incorporation assay

EdU single- or EdU and BrdU double-incorporation assay (1 nl and 10 mM, respectively) was performed as described in Supplementary Materials and methods. Injected embryos were incubated at 28.5°C till the desired time point for fixation in 4% paraformaldehyde for 2 h prior to cryo-sectioning. BrdU was detected by immunostaining and EdU incorporation by Alexa Fluor 488 Azide (Life Technologies, A10266).

### Flow cytometry analysis

Approximately 100 *Tg(lfabp: RFP)* zebrafish embryos were collected and fixed. Liver was then dissected under fluorescence microscope, followed by treatment with trypsin as previously described (Guan et al., 2016). For DNA content detection, cells were washed by phosphate-buffered saline (PBS) and re-suspended in PBS, then incubated with PI (50 μg/ml), and subjected to flow cytometry analysis of the cell cycle by using a BD FACS Calibur flow cytometer. Detailed protocols are described in Supplementary Materials and methods.

### RNA-seq and data analysis

Total RNA was extracted from WT and *bms1l^zju^*^*1*^ mutant embryos at 3dpf, respectively. RNA library construction and high-throughput sequencing were performed by Beijing Annoroad Gene Technology Company. Briefly, multiplexed libraries were sequenced for 150 bp at both ends using an Illumina HiSeq4000 platform. Clean reads were mapped to the zebrafish genome (Danio_rerio.GRCz10.84 from ENSEMBL). The threshold parameters for DEGs were an absolute fold change ≥2 and *P* <0.05. A GO enrichment analysis was performed using DAVID (version 6.8) (Huang et al., 2009).

### Immuno-TEM detection of RPA

Embryos were first fixed in 2.5% glutaraldehyde followed by three times washes in PBS. Liver was then dissected and fixed at 4°C overnight. Detailed protocols are described in Supplementary Materials and methods.

### ttf1a/ttf1b expression analysis

Total RNA was extracted from WT embryos at desired stages for generating cDNA by using M-MLV Reverse Transcriptase kit (Invitrogen, 28025-021). RT‒PCR products were obtained by using a pair of primers (F: 5′-CGACTCATTAAAGCGATGTATGA-3′; R: 5′-CTATTGATTAAAGCTGTTGTTCT-3′) perfectly matching both *ttf1a* and *ttf1b* sequences and cloned into the *pGEM-T* vector. Then, 96 individual *Escherichia coli* colonies were randomly picked for DNA sequencing to identify clones corresponding to *ttf1a* or *ttf1b* based on single-nucleotide polymorphisms between *ttf1a* and *ttf1b*.

### MO efficiency assay

MO was purchased from Gene Tools. The *ttf1*-MO (5ʹ-AATCTGACAGCATCTCATCCATCGT-3ʹ) was designed to target the TSC ATG region in both *ttf1a* and *ttf1b*. *ttf1a/ttf1b* TSC sequence was cloned upstream to the 5ʹ-end of the *EGFP* gene to construct the *pCS2^+^-TS-EGFP* plasmid for checking the efficiency of *ttf1*-MO (Supplementary Table S7).

### ChIP‒seq and ChIP‒qPCR

The ChIP‒seq experiment was performed based on previous zebrafish studies with modifications. ChIP‒qPCR primers were designed based on CHIP DNA peaks and listed in Supplementary Table S8. Detailed protocols are described in Supplementary Materials and methods. ChIP‒seq data GEO accession number is GSE176455.

### Assay for Ttf1a binding to peak-3

For DNA-binding assay, purified Flag-Ttf1a protein was mixed first with 5 fmol peak-3 DNA fragment with or without GTP (Thermol Scientific), and then with purified Bms1l-WT-HA, Bms1l-163-HA, or Bms1l-X-HA protein at 28°C for 1 h. The supernatant was collected for qPCR analysis, and the pellet was used as the protein sample for western blotting analysis. Detailed protocols are described in Supplementary Materials and methods.

### GTPase activity assay

The GTPase activity assay is based on checking the inorganic phosphate (Pi) level released from a phosphorylated substrate. This assay is performed under standard procedure of PiColorLock Phosphate Detection System (303-0030, Expedeon). Using purified Bms1l-WT-HA, Bms1l-163-HA, and Bms1l-X-HA with GTP (Thermol Scientific) to start the enzyme reaction for 5 and 60 min. The absorbance values at a wavelength between 590 and 650 nm were recorded. EDTA was used to stop the enzyme activity.

### Statistical analysis

For statistical analysis, comparisons were made using the Student’s *t*-test assuming a two-tailed distribution, with significance being defined as *P < 0.05, **P < 0.01, and ***P < 0.001. Details for each category of statistical analysis are provided in Supplementary Materials and methods.

## Supplementary material

Supplementary material is available at *Journal of Molecular Cell Biology* online.

## Supplementary Material

mjab074_Supplementary_Data
