# Supplementary material for: Nucleolar GTPase Bms1 displaces Ttf1 from RFB-sites to balance progression of rDNA transcription and replication
Source: J Mol Cell Biol. 2021 Nov 13;13(12):902–17. doi: 10.1093/jmcb/mjab074 (PMC8800533; doi:10.1093/jmcb/mjab074)
Supplement: mjab074_Supplementary_Data [file mjab074_supplementary_data.zip › Supplementary material.pdf]

Supplementary material

Nucleolar GTPase Bms1 displaces Ttf1 from RFB-sites to balance progression of rDNA transcription and replication

Yanqing Zhu[1,†], Yong Wang[2,†,*], Boxiang Tao[1], Jinhua Han[3], Hong Chen[1], Qinfang Zhu[1], Ling Huang[1], Yinan He[1], Jian Hong[4], Yunqin Li[4], Jun Chen[5], Jun Huang[3,*], Li Jan Lo[1,*], and Jinrong Peng[1,*]

[1] MOE Key Laboratory for Molecular Animal Nutrition, College of Animal Sciences, Zhejiang University, Hangzhou 310058, China

[2] Taizhou Hospital, Zhejiang University, Taizhou, 317000 China

[3] Life Sciences Institute, Zhejiang University, Hangzhou 310058, China

[4] Institute of Biotechnology, Zhejiang University, Hangzhou 310058, China

[5] College of Life Sciences, Zhejiang University, Hangzhou 310058, China

[†] These authors contributed equally to this work.

* Correspondence to: Jinrong Peng, E-mail: pengjr@zju.edu.cn; Yong Wang, E-mail: shorterw@sina.com; Jun Huang, E-mail: jhuang@zju.edu.cn; Li Jan Lo, E-mail: g0403022@zju.edu.cn

Supplementary Figures S1–S11

Supplementary Tables S1–S8

Supplementary Materials and methods

Fig. S1

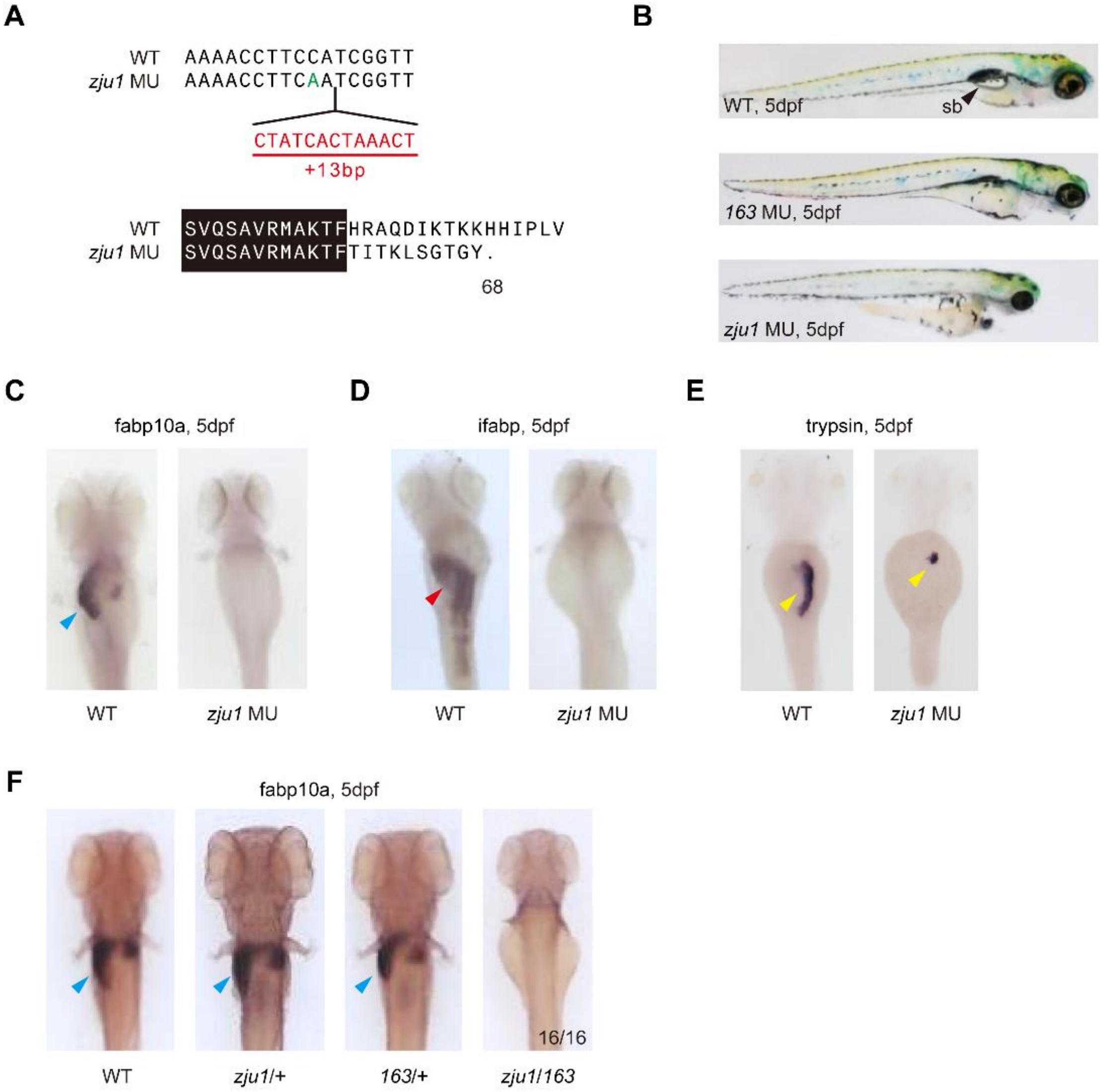

Supplementary Figure S1 Bms1l is essential for the development of digestive organs. (**A**) Generation of *bms1l*^{zju1} mutant (*zju1* MU). Upper panels show the one base substitution (green letter) and 13bp insertion (red letters) in the *bms1l*^{zju1} mutant (*zju1* MU). Lower panels compare amino acid sequences between the WT and Bms1l^{zju1} mutant proteins. ●, the translation of *bms1l*^{zju1} mRNA is stopped after Y. (**B**) Comparison of overall appearance among *bms1l*$^{sq163/sq163}$, *bms1l*$^{zju1/zju1}$ mutant and WT embryos at 5dpf. sb, swimbladder. (**C-E**) WISH using *fabp10a* (**C**), *ifabp* (**D**), *trypsin* (**E**) probes showing the hypoplastic phenotype of the liver (blue arrowhead) (**C**), intestine (red arrowhead) (**D**) and exocrine pancreas (yellow arrowhead) (**E**) in *bms1l*$^{zju1/zju1}$ mutant compared with WT at 5dpf. (**F**) WISH using an *fabp10a* probe to stain the liver (blue arrowhead) of progenies at 5dpf produced by crossing a *bms1l*$^{sq163/+}$ heterozygote with a *bms1l*$^{zju1/+}$ heterozygote, demonstrating that *bms1l*^{sq163} and *bms1l*^{zju1} are allelic.

Fig. S2

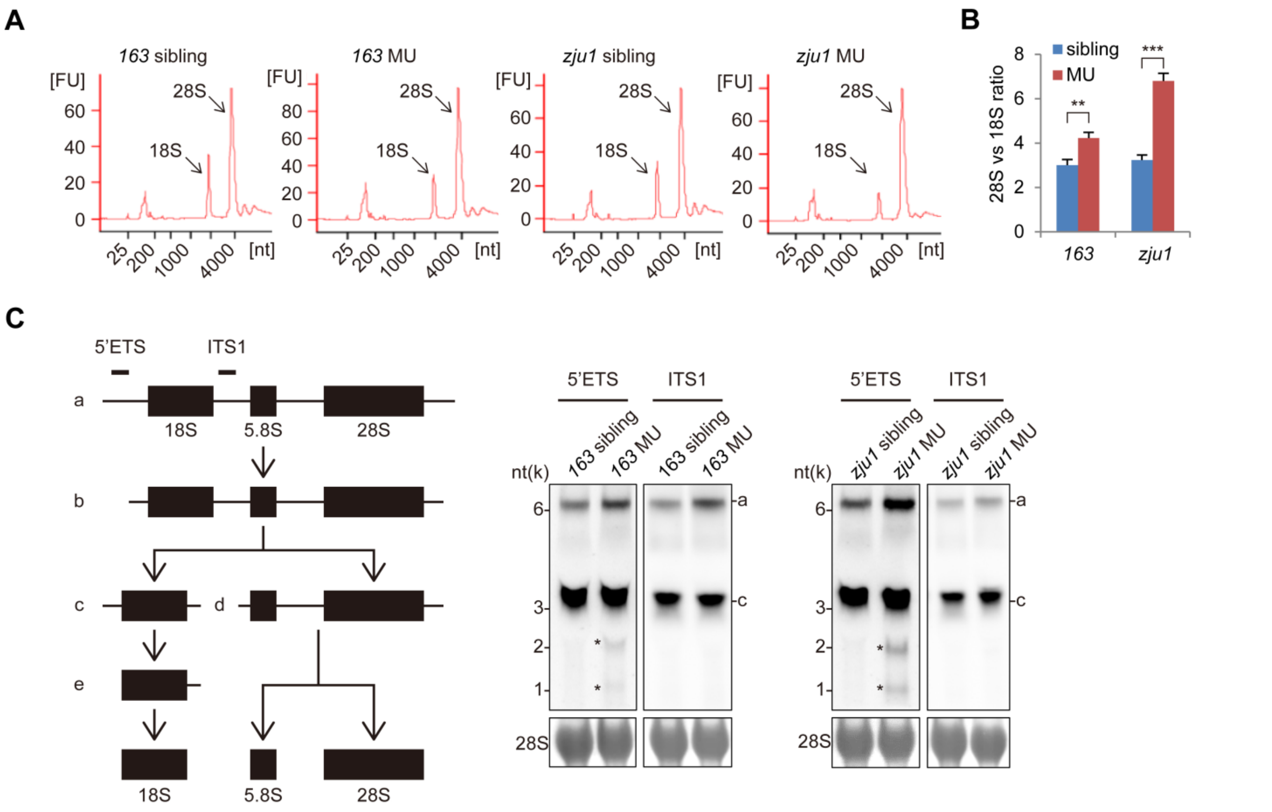

Supplementary Figure S2 Loss-of-function of Bms1l increases the 28S/18S ratio. (**A** and **B**) Quantifying the amount of 28S and 18S rRNA (**A**) showed that the ratios of 28S/18S in total RNA extracted from 5dpf-old *bms1l*^{sq163} and *bms1l*^{zju1} mutants were significantly higher than their respective siblings (**B**). (**C**) Northern blot analysis of pre-rRNA transcripts using the 5'ETS and ITS1 probes showed that the expression of pre-RNA was up-regulated in 5dpf-old *bms1l*^{sq163} (middle panels) and *bms1l*^{zju1} (right panels) mutants than their respective siblings. 5'ETS probe also detected two aberrantly intermediates in *bms1l*^{sq163} and *bms1l*^{zju1} mutants. Left panel: a diagram depicting the steps of pre-rRNA processing to produce mature 28S and 18S rRNAs and highlighting the 5'ETS and ITS1 probe locations. Asterisk, additional pre-rRNA processing products detected by the 5'ETS probe. 28S: 28S rRNA as the loading control.

Fig. S3

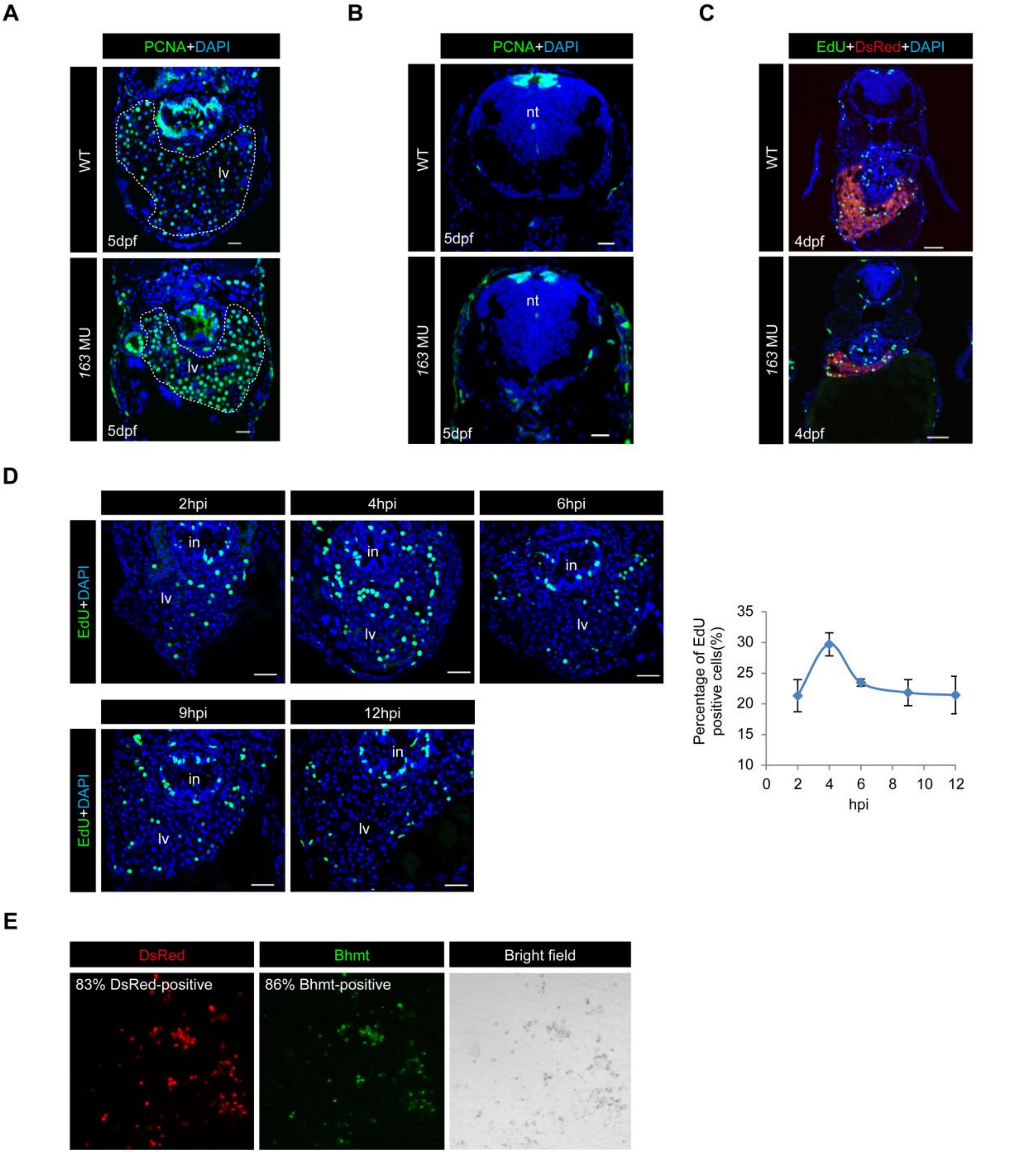

A
PCNA+DAPI
WT
lv
5dpf
163 MU
lv
5dpf
B
PCNA+DAPI
WT
nt
5dpf
163 MU
nt
5dpf
C
EdU+DsRed+DAPI
WT
4dpf
163 MU
4dpf
D
2hpi
4hpi
6hpi
EdU+DAPI
in
lv
9hpi
12hpi
Percentage of EdU positive cells(%)
35
30
25
20
15
10
0
2
4
6
8
10
12
hpi
E
DsRed
Bhmt
Bright field
83% DsRed-positive
86% Bhmt-positive

Supplementary Figure S3 Loss-of-function of Bms1l causes DNA partial over-replication and blocks the S to G2 transition. (**A** and **B**) Representative images of immunostaining of PCNA showing the significant increase in the percentage of PCNA-positive cells in *bms1l*$^{sq163/sq163}$ hepatocytes (**A**) but not in the neural tube (**B**) when compared with WT at 5dpf. Statistic data is shown in Figure 2A. Dotted frame in A: liver. Scale bars, 20µm. lv, liver; nt, neural tube. (**C**) Representative images of immunostaining of EdU showing the significant increase in the percentage of EdU-positive cells in *bms1l*$^{sq163/sq163}$ hepatocytes and a subtle increase in the mutant neural tube compared with WT at 4dpf, demonstrating active DNA biosynthesis in *bms1l*$^{sq163/sq163}$. For EdU staining (green), the fluorescent protein DsRed (red) expressed in *Tg(fabp10a:RFP)* reporter fish was used to define hepatocytes. Statistic data are shown in Figure 2B and C. Scale bar: 50µm. (**D**) Pulse-chase analysis of the incorporation of free EdU at 2, 4, 6, 9 and 12 hours post-injection at 4dpf by counting the number of EdU-positive cells in the WT liver (left image panels). No increase in the number of EdU labeled hepatocytes were recorded 6 hours after a single EdU injection (right panel). At each time point, 2615-5398 cells from at least three embryos were counted. in, intestine; lv, liver. Scale bar: 30µm. The values plotted represent mean ± SEM. (**E**) Detection of DsRed fluorescence and immunostaining of Bhmt to determine the identity of cells from the dissected liver from the embryos at 5dpf or 6dpf (in the *Tg(fabp10a:RFP)* genetic background). Such cells from *bms1l*$^{sq163/sq163}$ mutant and its siblings were subjected to DNA content analysis on a flow cytometry (Figure 2F). Corresponding bright field image was shown on the right. DAPI, staining the nuclei.

Fig. S4

A

#Samples	WT-1	WT-2	WT-3	*zju1* MU-1	*zju1* MU-2	*zju1* MU-3
Raw Bases Number	6276743100	6690529200	6305030400	6342002700	5427219600	5727208800
Raw Reads Length (bp)	150	150	150	150	150	150
Clean Bases Number	6258971100	6669428400	6298822500	6327615600	5417585700	5719536600
Clean Reads Length (bp)	150	150	150	150	150	150
Total reads	44588109	47803540	45406751	44998231	38607276	40222680
Mapping reads	37852177	41075909	39078940	38633325	32930176	33816005
Mapping rate (%)	84.89	85.93	86.06	85.86	85.3	84.07

B

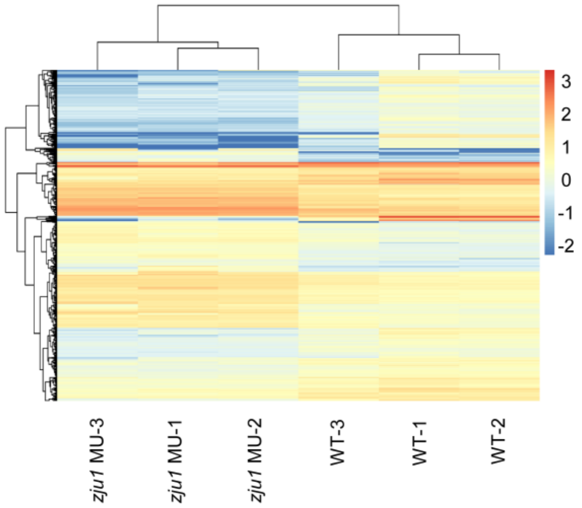

C

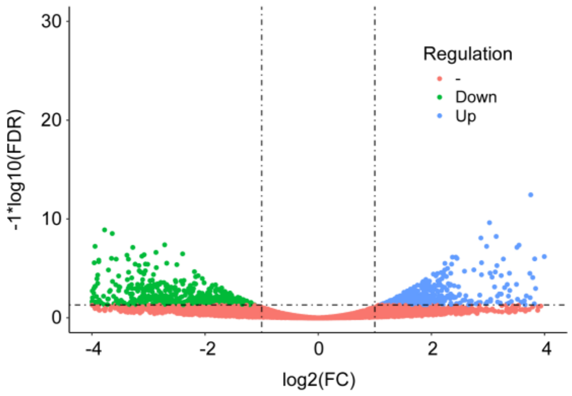

D

gene	gene ID	gene full name	log2FoldChange	P-value
lin52	777755	lin-52 DREAM MuvB core complex component	2.094984439	0.063
mcts1	394046	malignant T cell amplified sequence 1	1.703926807	***
lin54	560688	lin-54 DREAM MuvB core complex component	1.654071582	0.127
ccne1	30188	cyclin E1	1.233207972	*
nek8	171094	NIMA-related kinase 8	1.025734112	*

Supplementary Figure S4 Analysis of the RNA-seq data obtained from WT and *bms1l*^{zju1} mutant embryos at 3dpf. (**A**) Summary of the RNA-seq data (three biological repeats for WT and *bms1l*^{zju1}) including clean reads and mapping rates to the zebrafish genome. (**B**) Hierarchical Clustering analysis of the RNA-seq data between three WT samples and three *bms1l*^{zju1} mutant samples. (**C**) A volcano plot was constructed with the fold change value. (**D**) Identification of cell-cycle related genes upregulated ($\log_2 \geq 1$) in the *bms1l*^{zju1} mutant compared with WT at 3dpf. *, $p< 0.05$; ***, $p< 0.001$.

Fig. S5

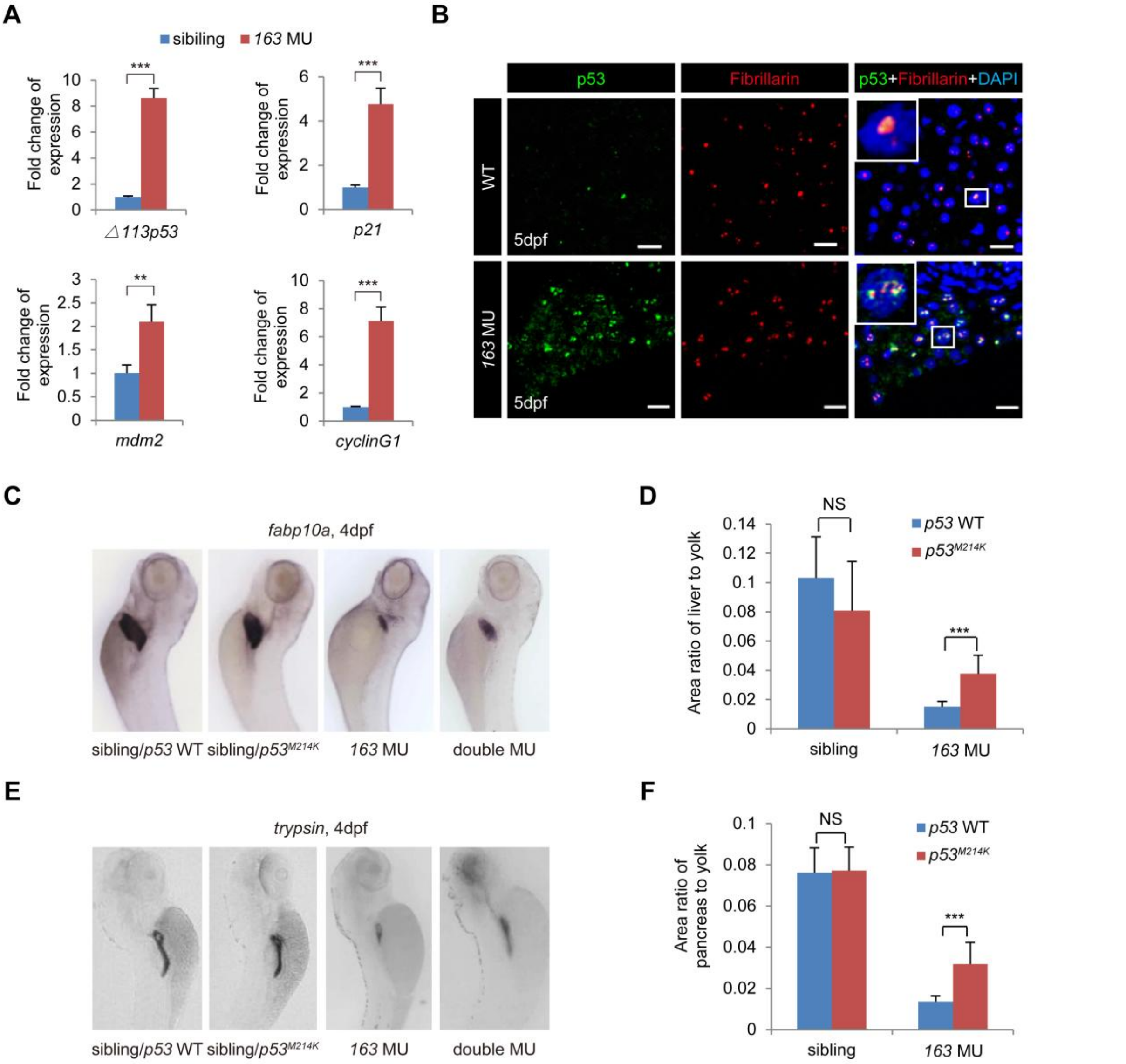

Supplementary Figure S5 Loss-of-function of Bms1l activates DNA damage response. (**A**) qPCR analysis showing the elevated expression of *Δ113p53, p21, mdm2* and *cyclinG1* in 5dpf-old *bms1l*$^{sq163/sq163}$ mutant compared with its siblings. The qPCR values were normalized against *GAPDH* and expressed as fold change of expression. (**B**) Immunostaining showing the obvious nucleolar enrichment of p53 and p53 isoforms in *bms1l*$^{sq163/sq163}$ hepatocytes compared with WT. Fibrillarin: nucleolar marker. DAPI: staining nuclei. Scale bars, 10μm. Insets: showing higher magnification image of a representative nucleus (boxed). (**C-F**) WISH using *fabp10a* (**C**) and *trypsin* (**E**) probe to compare liver (**D**) and exocrine pancreas (**F**) development among siblings, *p53*$^{M214K/M214K}$ single mutant, *bms1l*$^{sq163/sq163}$ single mutant and *bms1l*$^{sq163/sq163}$ *p53*$^{M214K/M214K}$ double mutant at 4 dpf. The liver and pancreas development was partially recovered in *bms1l*$^{sq163/sq163}$ *p53*$^{M214K/M214K}$ double mutant. 163 MU, *bms1l*$^{sq163/sq163}$. 10 to 13 double mutant embryos were examined after WISH. In **A**, **D** and **F**: the values plotted represent mean ± SEM. **, p< 0.01; ***, p< 0.001; NS, no significance.

Fig. S6

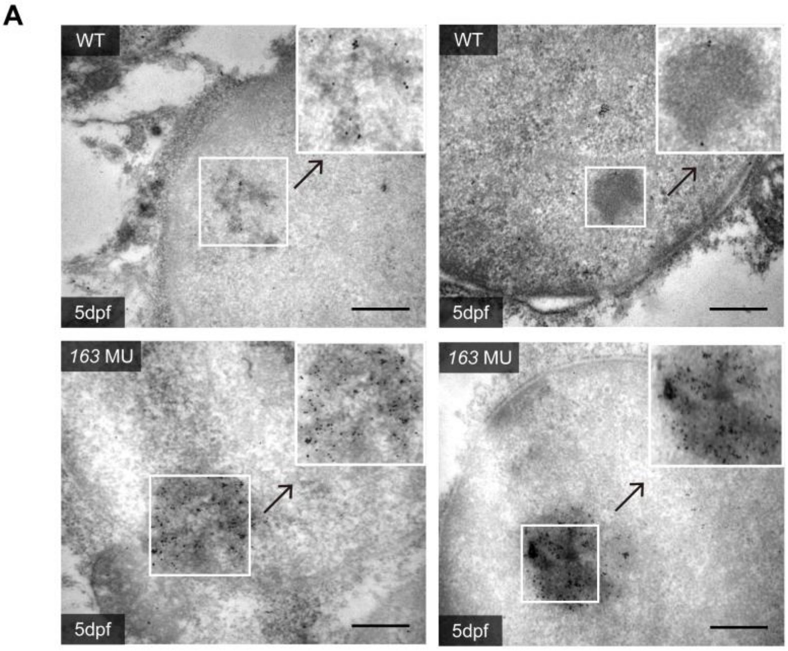

Supplementary Figure S6 Loss-of-function of Bms1l causes stalling of the rDNA replication fork progression. Representative images of immuno-TEM analysis of Rpa2 in WT and *bms1l*$^{sq163/sq163}$ mutant (images corresponding to Figure 4D and E) are shown. Boxed region, nucleoli. Inset, higher magnification image of the nucleolar region (boxed). Scale bars, 0.5μm.

Fig. S7

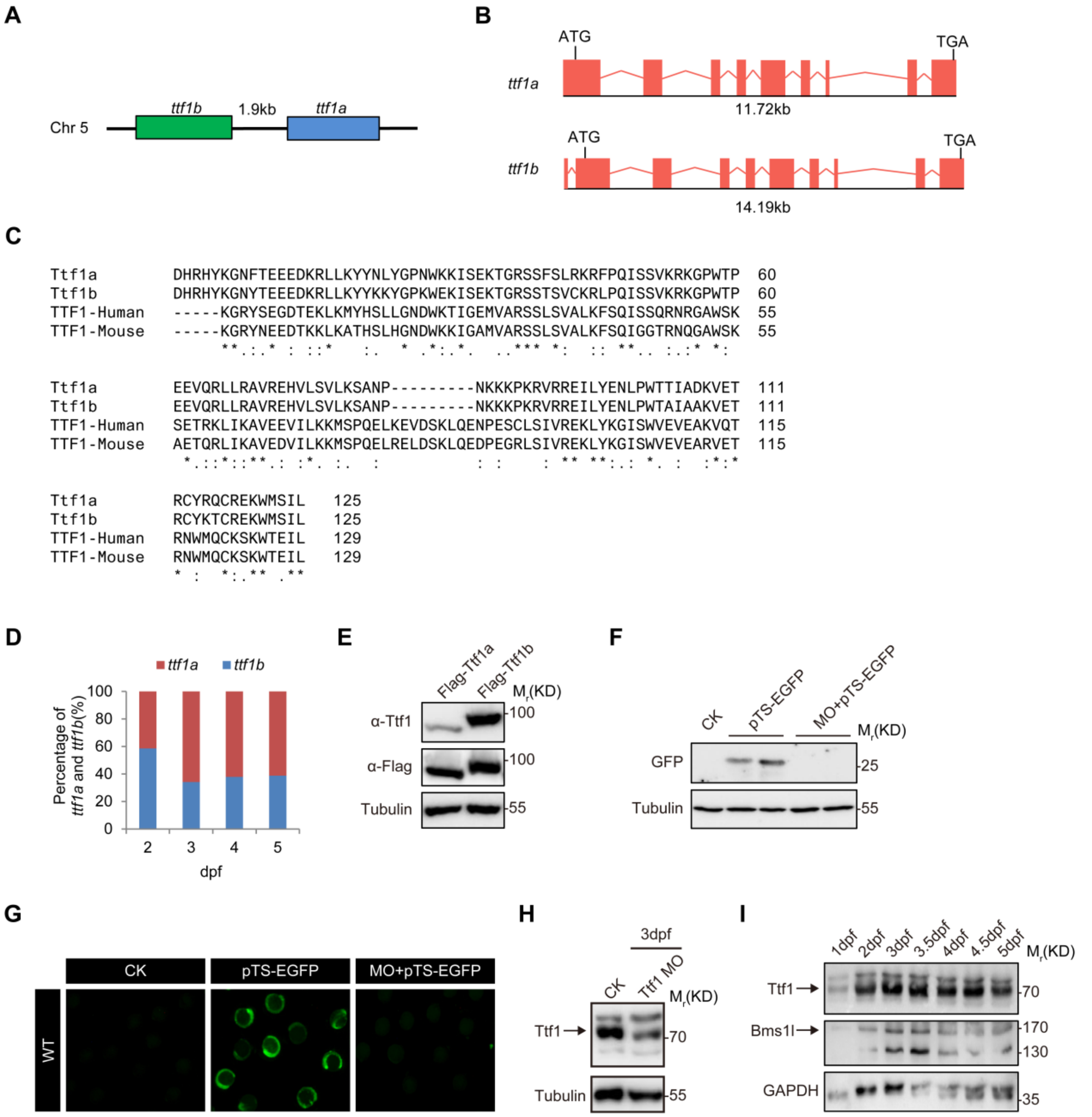
A
Chr 5
ttf1b
1.9kb
ttf1a
B
ATG
TGA
ttf1a
11.72kb
ATG
TGA
ttf1b
14.19kb
C
Ttf1a DHRHYKGNFTEEEDKRLLKYYNLYGPNWKKISEKTGRSSFSLRKRFPQISSVKRKGPWTP 60
Ttf1b DHRHYKGNYTEEEDKRLLKYYKKYGPKWEKISEKTGRSSTSVCKRLPQISSVKRKGPWTP 60
TTF1-Human -----KGRYSEGDTEKLKMYHSLLGNDWKTIGEMVARSSLSVALKFSQISSQRNRGAWSK 55
TTF1-Mouse -----KGRYNEEDTKKLKATHSLHGNDWKKIGAMVARSSLSVALKFSQIGGTRNQGAWSK 55
.:.* : ::* :. * .*:.*. ..* *: :: **.. :.:* *:
Ttf1a EEVQRLLRAVREHVLSVLKSANP---------NKKKPKRVRREILYENLPWTTIADKVET 111
Ttf1b EEVQRLLRAVREHVLSVLKSANP---------NKKKPKRVRREILYENLPWTAIAAKVET 111
TTF1-Human SETRKLIKAVEEVILKKMSPQELKEVDSKLQENPESCLSIVREKLYKGISWVEVEAKVQT 115
TTF1-Mouse AETQRLIKAVEDVILKKMSPQELRELDSKLQEDPEGRLSIVREKLYKGISWVEVEARVET 115
.::::**.: :*. :. : : : ** **::: *. : :*:*
Ttf1a RCYRQCREKWMSIL 125
Ttf1b RCYKTCREKWMSIL 125
TTF1-Human RNWMQCKSKWTEIL 129
TTF1-Mouse RNWMQCKSKWTEIL 129
* : *:.** .**
D
ttf1a ttf1b
Percentage of ttf1a and ttf1b(%)
100
80
60
40
20
0
2 3 4 5
dpf
E
Flag-Ttf1a
Flag-Ttf1b
Mr(KD)
α-Ttf1
100
α-Flag
100
Tubulin
55
F
CK
pTS-EGFP
MO+pTS-EGFP
Mr(KD)
GFP
25
Tubulin
55
G
CK
pTS-EGFP
MO+pTS-EGFP
WT
H
3dpf
CK
Ttf1 MO
Mr(KD)
Ttf1
70
Tubulin
55
I
1dpf
2dpf
3dpf
3.5dpf
4dpf
4.5dpf
5dpf
Mr(KD)
Ttf1
70
Bms1l
170
130
GAPDH
35

Supplementary Figure S7 Characterization of Ttf1 in zebrafish. (**A**) A schematic drawing showing that *ttf1a* (blue bar) and *ttf1b* (green bar) are located on chromosome 5 separated by a 1.9kb genomic DNA fragment. (**B**) Diagrams showing the genomic structure of *ttf1a* and *ttf1b*. The *ttf1a* gene spans an 11.72kb genomic DNA region and contains 9 exons and 8 introns. The *ttf1b* gene spans a 14.19kb genomic DNA region and contains 10 exons and 9 introns. Filled red box: exon; red line: intron. ATG: translation start codon; TGA: translation stop codon. (**C**) Alignment of the Myb-like DNA binding domain of Ttf1a and Ttf1b amino acid sequences together with TTF1 from human (NM_007344.4) and mouse (NM_009442.2). Myb-like DNA binding domain of zebrafish Ttf1a and Ttf1b share 90% identity. Myb-like DNA binding domain of zebrafish Ttf1a shares 33% identity with both human and mouse TTF1. (**D**) A *ttf1a* and *ttf1b* common primer pair were used to perform RT-PCR. The RT-PCR products were cloned into the pGEM-T vector. Individual clones were picked and sequenced to identify *ttf1a* and *ttf1b* specific sequences. The result revealed that *ttf1a* transcripts were relatively abundant in the 3dpf-, 4dpf- and 5dpf-old embryos. (**E**) Western blot analysis showing the detection of both Ttf1a and Ttf1b by a Ttf1 monoclonal antibody and the Flag-tag antibody, respectively. *Flag*-tagged *Ttf1a* and *Ttf1b* plasmids in the *pCS2* vector were transfected into 293T cells. Total protein was harvested 48 hours post transfection and was subjected to western blot analysis. Tubulin: loading control. (**F** and **G**) Analysis of the effectiveness of the *ttf1*-MO which specifically targets both *ttf1a* and *ttf1b* at around their translation start codons. The translation start codon ATG region of *ttf1a/ttf1b* was cloned in-frame upstream to the reporter gene *EGFP* to generate the plasmid *pTS-EGFP*. The *pTS-EGFP* plasmid was injected alone or co-injected with *ttf1*-MO into fertilized eggs at one-cell stage. The expression of EGFP in the injected embryos was examined by a western blot analysis of total proteins extracted from the injected embryos at 12hpf (**F**) and was also visualized under a fluorescence microscope (**G**). (**H** and **I**) The identity of the endogenous Ttf1 was determined by its downregulation in the morphants after ttf1-MO injection (**H**). Western blot was then performed to analyze the expression of Ttf1 and Bms1l in 1dpf- to 5dpf-old embryos (**I**). GAPDH: loading control.

Fig. S8

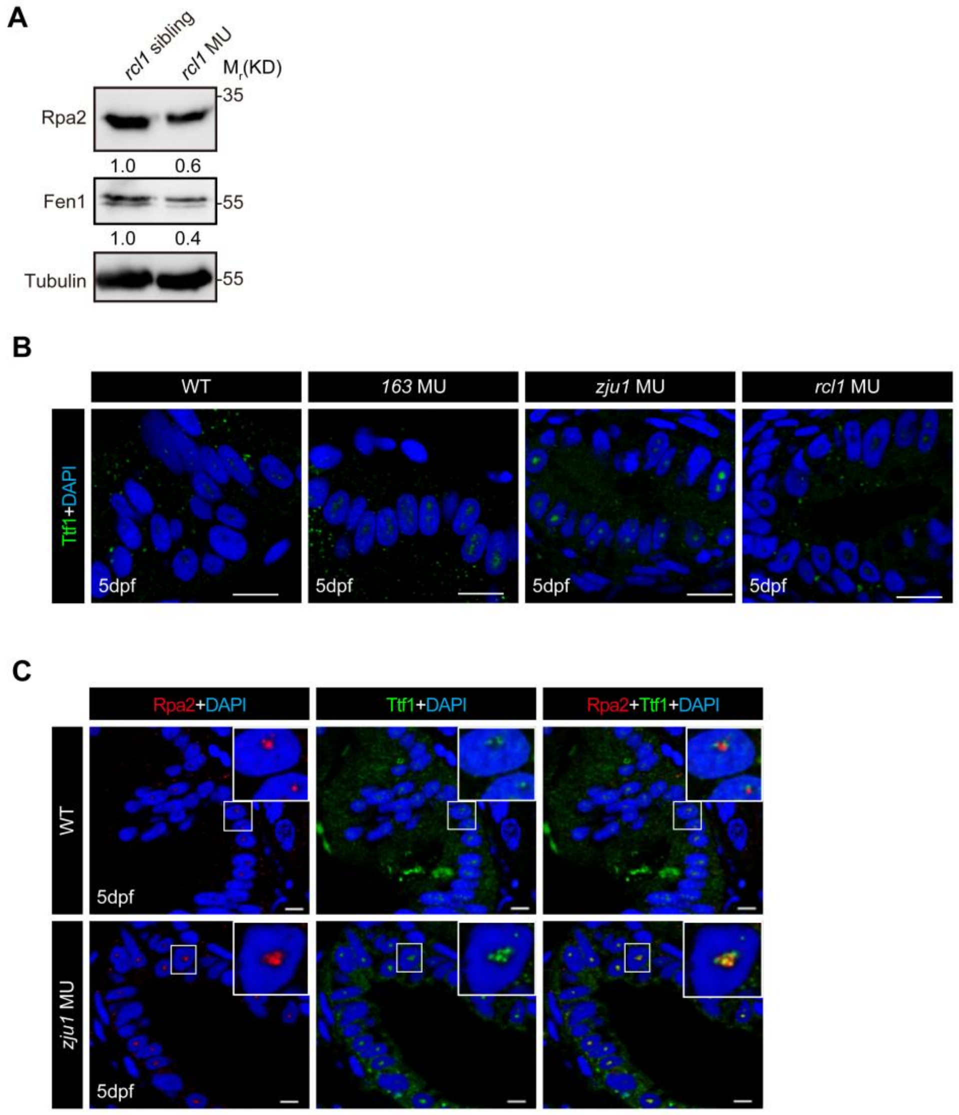

Supplementary Figure S8 Loss-of-function of Bms1l causes accumulation of Ttf1 in the nucleolus. (**A**) Western blot analysis did not reveal obvious difference for the expression of Rpa2, and Fen1 between *rcl1*$^{-/-}$ (*rcl1* MU) and corresponding siblings at 5dpf. Tubulin: loading control. (**B**) Immunostaining of Ttf1 showed that Ttf1 was enriched in the nucleolus of gut epithelia in *bms1l*$^{sq163/sq163}$ and *bms1l*$^{zju1/zju1}$ but not *rcl1*$^{-/-}$ mutants. Scale bars, 10μm. Statistic data was shown in Figure 5C. (**C**) Co-immunostaining of Ttf1 and Rpa2 showed a significantly higher ratio of the nucleoli displaying intervening signals of Ttf1 and Rpa2 in the *bms1l*$^{zju1/zju1}$ mutant gut epithelia. Scale bars, 5μm. Statistic data was shown in Figure 5D. Inset: showing the higher magnification image of a representative nucleus (boxed).

Fig. S9

A

```
p1    -------------------TGGGCCTCGACGAGGCTCGTGCAGTGTTCCAAAGAGCAAGA    41
p2    ---------------------------------------------------AAAAAGAGG    9
p3    GACCAGTGGCTCGCTTTCTCTTGAGAGTGAGGGGAATCGGCTTGACTCTACAAAAAGAGG    60
p4    ---GTTGTACAGTGCCTCTCTTGAGAGTGAGGGGAATCGGCTTGACTCTC-TAAAAAAGG    56
                                                           *   **

p1    AAAGACCCACCATCCCCCAAGTGAGTGTTGGAACCACCAGTGACTCTTTGCCTATTCTCT    101
p2    ACAGACCCACCATCCCCCAAGTGAGTGTTGGAACCACCAGTGACTCTTTGCCTTTTCTCT    69
p3    ACAGACCCACCATCCCCCAAGTGAGTGTTGGAACCACCAGTGACTCTTTGCCTTTTCTCT    120
p4    ACAGACCCACCATCCTCCAAGTGAGTGTTGGAACGACCAGTGACTCTTTGCCTATTCTCT    116
      * ************* ****************** ****************** ******

p1    CTTTCTAACCC----------CCCCTTTCTTACAGGGTGAGCTGAATCGGCTTGACTTTG    151
p2    CTTTCTAAACCCCCCCCCCCCCCCCCTTCTTACAGGGTGAGCTGAATCGGCTTGACTTTG    129
p3    CTTTCTCTTTCTTC-----TCTCTCCTTCTTACAGGGTGAGCTGAATCGGCTTGACTTTG    175
p4    CTTTCTAACCC----------CCCCTTTCTTACAGGGTGAGCTGAATCGGCTTGACTTTG    166
      ******    *           * * **********************************

p1    ACAAAAATGGAGGAAACCCACCACCCATAGACAGAGAGCAGGAAACCTATCCCCCAGCGT    211
p2    ACAAAAATGGAGGAAACCCACCACCCATAGACAGAGAGCAGGAAAGC-------------    176
p3    ACAAAAATGGAGGAAACCCACCACCCATAGACAGAGAGCAGGAAACCTA-----------    224
p4    ACAAAAATGGAGGAAACCCACCACCCCTAGACAGAGAGCAGGAAAGCCATCCCCC-----    221
      ************************** ****************** *

p1    GAG    214
p2    ---    176
p3    ---    224
p4    ---    221
```

B

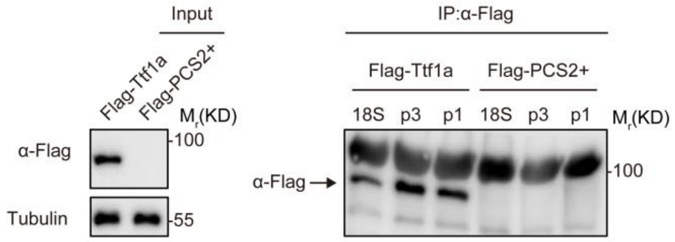

C

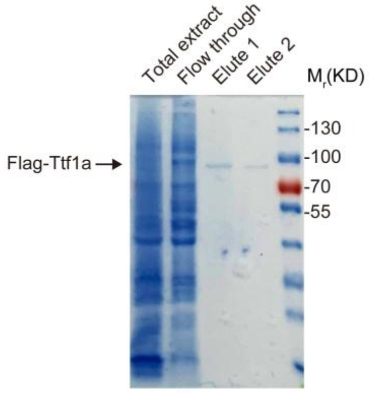

Supplementary Figure S9 Binding of Ttf1 to target sequence downstream of the rDNA gene is GTP-dependent. (**A**) Sequence alignment of the four Ttf1-binding sites peak-1 to peak-4 (p1 to p4) downstream of the 3'-end of the rDNA gene. (**B**) Western blot analysis of Flag-Ttf1a in the input (left) and in the Co-IP products (right) which were used as the substrate for ChIP-qPCR analysis of the binding of Ttf1a to peak-1 (p1), peak-3 (p3), 18S and 28S rRNA as shown in Figure 5F. Total protein extracted from the 293T cells expressing Flag-Ttf1a was mixed with peak-1, peak-3 and 18S rDNA, respectively, and the mixture was subjected to Co-IP using a Flag-tag specific antibody. (**C**) Coomassie blue stained protein gel to show the purified Flag-Ttf1a using Flag-antibody conjugated agarose beads, and elute with Flag-peptide (lanes 3 and 4). This purified Flag-TTF1s protein was used for DNA binding assay showed in Figure 5H and I. Lane 1: total protein extracts; lane 2: flow through.

Fig. S10

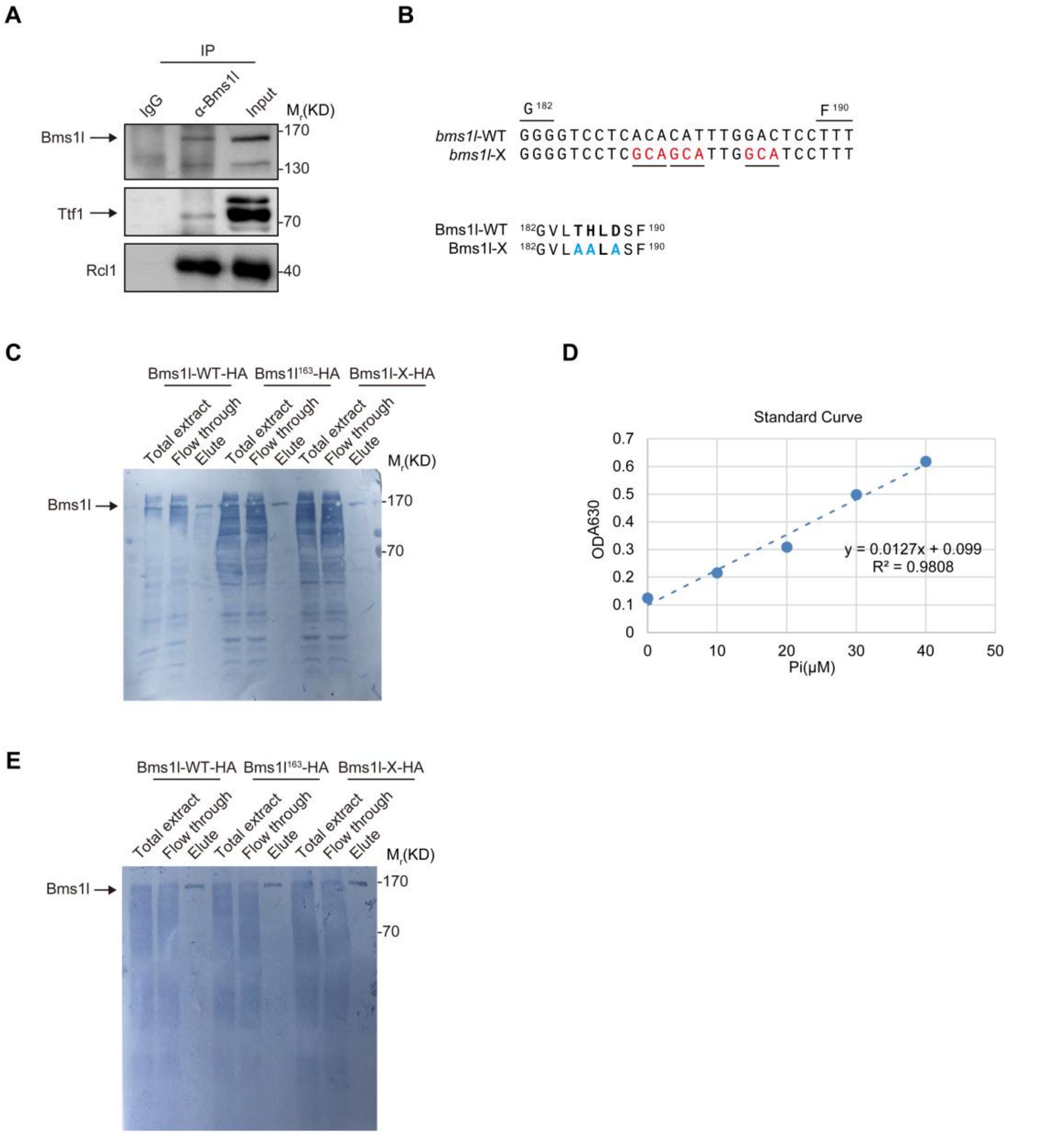

A
IP
IgG
α-Bms1l
Input
Mr(KD)
Bms1l
Ttf1
Rcl1
-170
-130
-70
-40
B
G 182
F 190
bms1l-WT GGGGTCCTCACACATTTGGACTCCTTT
bms1l-X GGGGTCCTCGCAGCATTGGCATCCTTT
Bms1l-WT 182GVLTHLDSF190
Bms1l-X 182GVLAALASF190
C
Bms1l-WT-HA
Bms1l163-HA
Bms1l-X-HA
Total extract
Flow through
Elute
Mr(KD)
Bms1l
-170
-70
D
Standard Curve
ODA630
Pi(μM)
y = 0.0127x + 0.099
R² = 0.9808
0
0.1
0.2
0.3
0.4
0.5
0.6
0.7
0
10
20
30
40
50
E
Bms1l-WT-HA
Bms1l163-HA
Bms1l-X-HA
Total extract
Flow through
Elute
Mr(KD)
Bms1l
-170
-70

Supplementary Figure S10 Both WT Bms1l and mutant Bms1l^{163} protein interact with TTF1 but only WT Bms1l displaces Ttf1 from the RFB sites. (**A**) Co-IP analysis showed that the endogenous Bms1l interacted with Ttf1. Total proteins were extracted from 3dpf-old WT embryos and were subjected to Co-IP using the Bms1l antibody. (**B**) Alignment of DNA sequence (upper panel) and amino acid sequence (lower panel) to show the base changes (in red) in *bmsl1-X* and amino acid changes (in blue) in Bms1l-X. (**C**) Coomassie blue stained protein gel to show the purified HA-tagged Bms1l-WT (lane 3), Bms1l-163 (lane 6) and Bms1l-X (lane 9) using HA-antibody conjugated agarose beads, and elute with HA- peptide. These purified proteins were used to determine their GTPase activities as shown in Figure 6D. (**D**) Absorbance value (A630) at 5min versus free Pi concentration was used to obtain the standard curve for GTPase activity assay. (**E**) Coomassie blue stained protein gel to show the purified HA-tagged Bms1l-WT (lane 3), Bms1l-163 (lane 6) and Bms1l-X (lane 9) using HA-antibody conjugated agarose beads, and elute with HA- peptide. These purified proteins were used to determine their ability to disassociate the Ttf1-peak3 complex as shown in Figure 6E.

Fig. S11

A

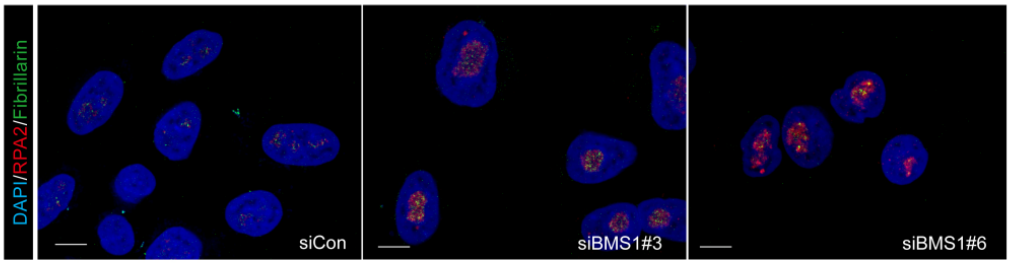

B

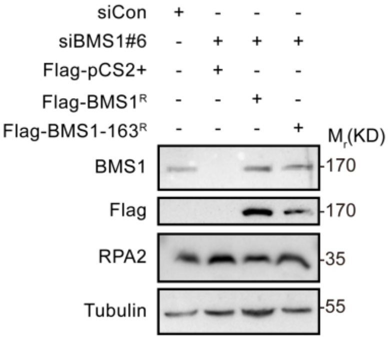

Supplementary Figure S11 Knockdown of human BMS1 causes DNA over-replication and replication-fork stall. (**A**) images of co-immunostaining of RPA2 and Fibrillarin in HeLa cells treated with siCon, siBMS1#3 and siBMS1#6 (96hrs post-transfection). DAPI: staining nuclei. Scale bars, 5μm. (**B**) Western blot analysis of BMS1, Flag-BMS1^R and Flag-BMS1-163^R using BMS1 antibody or Flag-tag antibody, and of RPA2 using RPA2 antibody in Hela cells pre-treated (24hrs) with siCon or siBMS1#6.

Supplementary Table S3 List of the top 20 significantly enriched items in the BP_down (downregulated genes) category.

Description	Count	%	$log_{10}P$	$log_{10}q$	Gene list
Visual phototransduction	9	3.1	-7.38	-3.31	rho, opn1sw1, rbp3, gnat1, guca1c, guca1d, cyp4v8, rcvrna, saga, rcvrn2, grk1b
Organic acid metabolic process	27	9.31	-6.3	-2.93	pcxb, agxtb, cyp2ad2, tdo2b, kmo, pah, hyi, hpda, got2a, aldh6a1, mpc1, acsl1b, acat1, slc27a2a, me3, asah2, cyp2u1, agmat, cyp4v8, cyp4v7, cyp2k19, mlsl, elovl2, pm20d1.2, gls2a, cyp2p8, prodhb
Phase I - Functionalization of compounds	9	3.1	-6.29	-2.93	cyp2ad2, mao, aoc1, cyp2u1, cyp4v8, ces3, ncoa1, tbxas1, cyp2p8, ahcy, dpep1, ephx2, pla2g1b, cpb1, cyp3a65, cyp2k19
Cone photoresponse recovery	4	1.38	-6.06	-2.77	rcvrn2, grk1b, rcvrna, rcvrn3
Response to light stimulus	11	3.79	-4.9	-1.91	rho, opn1mw1, opn1sw1, gnat1, rcvrn2, grk1b, rgra, slc45a2, trpm1b, rcvrna, rcvrn3
Cellular aldehyde metabolic process	6	2.07	-4.45	-1.71	agxtb, bco2l, hyi, bco1l, mlsl, bco2a
Metabolism of lipids	17	5.86	-4.43	-1.71	cyp2ad2, pon2, scp2a, fabp6, acat1, asah2, fabp1b.1, cyp2u1, enpp7.1, ces3, ncoa1, pla2g1b, elovl2, dpep1, faah2b, tbxas1, cyp2p8
Glyoxylate metabolic process	3	1.03	-4.13	-1.47	agxtb, hyi, mlsl
Phenylalanine metabolism	4	1.38	-4.03	-1.43	pah, hpda, mao, got2a
Amine metabolic process	6	2.07	-3.89	-1.32	tdo2b, kmo, mao, aoc1, agmat, oaz2a
Carbon metabolism	9	3.1	-3.71	-1.18	pcxb, agxtb, fbp1a, got2a, aldh6a1, acat1, me3, tkta, tkfc
Regulation of lipid transport	3	1.03	-3.37	-0.91	scp2a, abcg1, asip2b
The canonical retinoid cycle in rods (twilight vision)	3	1.03	-3.01	-0.67	rho, rbp3, cyp4v8
Cellular response to estrogen stimulus	4	1.38	-2.92	-0.6	agxtb, ucp1, pah, aqp12
PPAR signaling pathway	5	1.72	-2.77	-0.48	scp2a, fabp6, cd36, acsl1b, cyp8b1, ncoa1
Metabolism of amino acids and derivatives	8	2.76	-2.46	-0.26	agxtb, ahcy, got2a, aldh6a1, acat1, slc45a2, oaz2a, si:ch211-286f9.2
Retinol metabolism	4	1.38	-2.45	-0.26	rdh8a, bco1l, rdh8b, cyp3a65
Response to yeast	3	1.03	-2.21	-0.08	kmo, sc5d, slc15a2
Negative regulation of endopeptidase activity	4	1.38	-2.08	0	serpinf2a, ahsg2, ahsg1, timp4.3
Oxidative stress response	3	1.03	-2.07	0	gstt1b, mao, sod3b

Supplementary Table S4 List of the top 20 significantly enriched items in th BP_up (upregulated genes) category.

**Description**	**Count**	**%**	**$log_{10}P$**	**$log_{10}q$**	**Gene list**
ncRNA metabolic process	49	16.3	-31.08	-27.02	vars1, snu13b, ddx18, rrp36, ybey, wdr36, gnl3, rpl7l1, mphosph10, esf1, rrp15, iars1, heatr1, rars1, rrs1, cars1, wdr75, dcaf13, exosc4, qtrt1, ddx52, rpf1, imp4, riok3, sars1, tars1, exosc9, thg1l, farsb, trmt13, rrp7a, exosc7, fcf1, mrto4, trmt1, trmu, dph3, yars2, tyw1, trmt6, pus10, nars1, lars1b, ptcd1, wdr4, dkc1, emg1, farsa, rrp8, coil, npm1a, ruvbl1, ppan, prmt7, heatr3, mcts1, bxdc2, ptges3b, nop16, mpv17l2, polr1d, mrpl10, snrpd2, cpsf3, snrnp25, dnttip2, magoh
tRNA metabolic process	24	8	-16.39	-13.18	vars1, iars1, rars1, cars1, qtrt1, sars1, tars1, exosc9, thg1l, farsb, trmt13, exosc7, trmt1, trmu, dph3, yars2, tyw1, trmt6, pus10, nars1, lars1b, ptcd1, wdr4, farsa, dkc1, emg1, prmt1, prmt5, prmt7, prmt6, ndufaf7, dph5, rrp8
tRNA aminoacylation for protein translation	11	3.67	-10.84	-7.77	vars1, iars1, rars1, cars1, sars1, tars1, farsb, yars2, nars1, lars1b, farsa, igf2bp3, eif2s1a, brf1a, drg1, mcts1, eif2b2, eif5a2, mrpl43, exosc9, dph5, mrpl10, exosc7, dph3, mrpl27, chchd1, eif2b5, tsfm, pde12, gemin5, magoh, agk, asns, sptssa, odc1, hpdl, cyp26a1, ptgs2a, hsd17b12b, lias, hk2, ptges3b, ptges, ptgs2b, aldh1l2
Ribosomal large subunit biogenesis	12	4	-9.71	-6.83	npm1a, ppan, snu13b, ddx18, rpl7l1, rrp15, rrs1, heatr3, bxdc2, rpf1, nop16, mrto4
Protein folding	14	4.67	-7.74	-5	hsp70.3, cct3, unc45b, ahsa1b, pfdn2, hspa5, nudc, canx, ptges3b, fkbp1ab, grpel1, hspa14, dnlz, dnajc25, hspb8
Ribosomal small subunit biogenesis	9	3	-6.49	-3.83	npm1a, snu13b, rrp36, heatr1, rrs1, dcaf13, ddx52, riok3, rrp7a
Peptidyl-arginine methylation	5	1.67	-6.1	-3.52	prmt1, prmt5, prmt7, prmt6, ndufaf7
Maturation of 5.8S rRNA	6	2	-5.12	-2.59	gnl3, rrp15, rrs1, rpf1, exosc9, exosc7, exosc4, ptcd1, dkc1, rrp36, cpsf3, tent5c, heatr1, slirp, mrto4, pde12, magoh, moxd1, aldh1l2, igf2bp3, eif5a2, dph5, dph3, coil, snu13b, prmt7, snrpd2, snrnp25, ube2d2l, adam8a, hspa5, canx, psmd8, usp4, lonp1, psmb3, yme1l1a, adam8b
Regulation of HSF1-mediated heat shock response	7	2.33	-5.06	-2.55	ptgs2a, hsd17b12b, lias, asns, ptges3b, ptges, ptgs2b
Prostaglandin biosynthetic process	4	1.33	-4.36	-1.95	ptgs2a, ptges3b, ptges, ptgs2b
Hypertrophy model	4	1.33	-4.22	-1.83	ptgs2a, ptges3b, ptges, ptgs2b

Ribonucleoprotein complex assembly	10	3.33	-4.13	-1.75	coil, ruvbl1, ppan, prmt7, mcts1, bxdc2, ptges3b, rrp7a, snrpd2, mrto4, mpv17l2, mef2ca, brf1a, tcap, nup107, lonp1, coa5, ndufaf7
Mitochondrion organization	12	4	-3.5	-1.22	phb, agk, slirp, timm50, atad3, timm10, grpel1, lonp1, coa5, ndufaf7, dnlz, yme1l1a, heatr3, pex5, ipo9, nup107, mon1a, tomm22, xpo6, arl14, rab3da
Nucleocytoplasmic transport	8	2.67	-3.41	-1.17	npm1a, xpo6, anp32a, rrs1, heatr3, ipo9, thoc3, nup107, igf2bp3, eif5a2, magoh
Myosin filament organization	3	1	-3.33	-1.12	mef2ca, tcap, hspb11, shha, cyp26a1, unc45b, nppa, prmt5, bag3, smyd5, sil1
Regulation of translational elongation	3	1	-3.33	-1.12	eif5a2, dph5, dph3
RNA polymerase	3	1.33	-2.93	-0.81	heatr1, cavin1a, dhx33
Multicellular organismal response to stress	3	1	-2.87	-0.76	hsd11b2, fosab, prl
Regulation of sprouting angiogenesis	3	1	-2.87	-0.76	iars1, sars1, tars1
Inflammatory response	10	3.33	-2.81	-0.71	ptgs2a, adam8a, mmp9, ptgs2b, gbp3, tnfaip6, cxcl18b, ccl39.6, cxcl8a, adam8b, mmp13a, c3a.1, smyd5, usp4, c7a, mapkapk3, c4b, bcl6ab, tcima, socs3a, f3b, hbegfa, serpine1, g3bp1, prmt7

Supplementary Table S5 Statistics for ChIP-Seq data using the Ttf1 antibody.

Item	Data
Total Clean Reads	45681870
Unique Mapped Reads	29654670
Unique Mapped Ratio (%)	64.92
Multi Mapped Reads	9800496
Multi Mapped Ratio (%)	21.45
Mapped Ratio (%)	86.37

Supplementary Table S6 ChIP-Seq identification of Ttf1-binding sites in the zebrafish genome.

Chr	Start	End	Length	Fold change	Description	Input IP
chr2	15006986	15007323	338	7.87488	Undefined	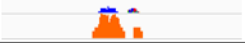
chr2	19780201	19780848	648	8.21625	Undefined	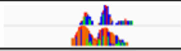
chr2	19826279	19827017	739	4.85732	Undefined	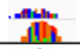
chr2	14338648	14338966	319	4.54573	Undefined	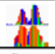
chr4	76322610	76325961	3352	5.73664	NLRC3-like	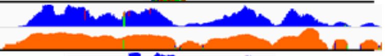
chr4	76319958	76321447	1490	3.20957	NLRC3-like	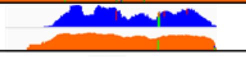
chr4	28576176	28576472	297	7.61758	Undefined	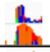
chr4	28604956	28605470	515	6.76305	Undefined	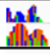
chr4	28581554	28581960	407	6.26332	Undefined	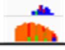
chr4	28596379	28597684	1306	5.95543	Undefined	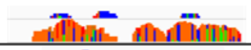
chr4	28572876	28573899	1024	5.12527	Undefined	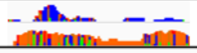
chr5	829028	830853	1826	4.11776	Downstream of rDNA	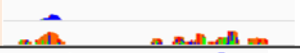
chr7	30506067	30507894	1828	10.43191	mphosph10	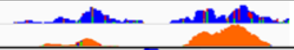
chr7	52740056	52740575	520	9.39672	Undefined	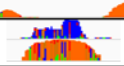
chr7	64181671	64181987	317	5.97667	Undefined	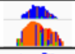
chr7	52742932	52743397	466	5.81208	Undefined	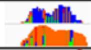
chr7	64183504	64184933	1430	5.69647	Undefined	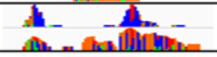
chr8	34866289	34867130	842	6.50198	Undefined	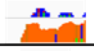
chr8	12874297	12874588	292	4.56488	Undefined	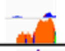
chr10	16842664	16842953	290	8.54181	Undefined	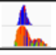
chr10	16840574	16841595	1022	6.45816	Undefined	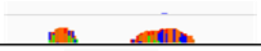
chr10	16836101	16836684	584	4.45713	Undefined	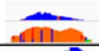
chr11	17430424	17431647	1224	7.03834	Undefined	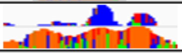
chr14	19788730	19789339	610	8.60394	Undefined	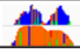
chr16	26068364	26068717	354	8.7311	Undefined	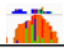
chr16	26005945	26006883	939	5.97344	Undefined	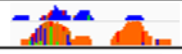
chr16	15767949	15768726	778	2.79131	Undefined	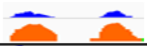
chr17	39991158	39992207	1050	7.29315	Undefined	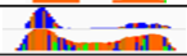
chr20	6461100	6461819	720	4.0594	Trappc8	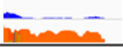
chr20	55336087	55338196	2110	16.38265	Undefined	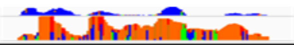
chr20	17121450	17121736	287	9.26523	Undefined	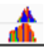
chr21	25811993	25812247	255	7.87726	Undefined	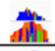
chr21	25828231	25828864	634	6.5949	Undefined	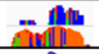
chr22	39221276	39222287	1012	2.57131	Undefined	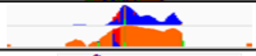
chr23	16245963	16247254	1292	5.14046	Undefined	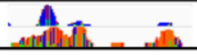

Note: ChIP-seq data GEO accession number: GSE176455. Corresponding chromatin peaks are shown on the right. Blue: input; orange: Ttf1 antibody IP product.

Supplementary Table S7 Primer sequences for plasmid construction and siRNA for targeting human BMS1.

Primer	Sequence (5'-3')
HA-Bms1l-F	GTACGGATCCGCCACCATGTACCCATACGATGTTCCAGATTACGCTG AGAGGAAAGAGCAGAAGCG
HA-Bms1l-R	ACCGCTCGAGTCAATTGTCCTTGGATGTTCCCTT
Flag-Ttf1-F	ATGCGATATCGATGAGATGCTGTCAGATTC
Flag-Ttf1-R	GCATCTCGAGCTATTAATTAAAGCTGTTGTT
EGFP-TS-F*	GATCCCATCGATTCG**ACG<u>ATG</u>GATGAGATGCTGTCAGATT**CAGTGA GCAAGGGCGAGGAGCT
EGFP-TS-R	GCTCGAGAGGCCTTGTTACTTGTACAGCTCGTCCA
Flag-BMS1R-F	TGGAGGCCCCCCATGACTGGGACCTTGAAGAAGTAATGAACAGTATCA GAGATTGCTT
Flag-BMS1R-R	AAGCAATCTCTGATACTGTTCATTACTTCTTCAAGGTCCCAGTCATGGG GGGCCTCCA
**siRNA targeting human BMS1**	
siBMS1#3	AGUUUCCGUCGUUUGAUGC
siBMS1#6	AUAACCUCCUCUAAAUCCC
siControl	UUCAAUAAAUUCUUGAGGUUU

* Bold letters are the ttf1-MO target sequence. Translation start codon ATG is underlined.

Supplementary Table S8 Primer sequences for qPCR.

**Primer**	**Sequence (5’-3’)**
45S P1-F	CCACGAGTCTTTGGGTTCC
45S P1-R	CGCTCCACCAACTAAGAACG
45S P2-F	GGGTGCAGATCTTGGTGGTA
45S P2-R	GATTTGAACCCGACTCCCTT
ubf-F	GACGGCGAACCTAAGAAACC
ubf-R	TTCTCCTCTGCAATGCGTTT
tif-1a-F	AGCAATGGTTCATCCTGTGG
tif-1a-R	CAGGAAGCATGAGTGGGAAG
taf1b-F	CAAATCTGAAGCGCAAGTCC
taf1b-R	TCTGAACCCTCCTCCTCTCC
⊿113p53-F	ATATCCTGGCGAACATTTGGAGGG
⊿113p53-R	CCTCCTGGTCTTGTAATGTCAC
p21-F	GAAGCGCAAACAGACCAACAT
p21-R	GCAGCTCAATTACGATAAAGA
mdm2-F	CTCGCAGTGAGGGCAGTGAAG
mdm2-R	TCTAGGCACGTAGCGGGAAGG
cyclinG1-F	GCCCTTTACAGTCCAGCCCAAATC
cyclinG1-R	CTGTGCCTCAAGCCTCTCGATGTA
cyclinE1-F	TCCCGACACAGGTTACACAA
cyclinE1-R	TTGTCTTTTCCGAGCAGGTT
cdk2-F	CAGCTCTTCCGGATATTTCG
cdk2-R	CCGAGATCCTCTTGTTTGGA
ChIP-p1-F	GTGTTGGAACCACCAGTGAC
ChIP-p1-R	CTGTCTATGGGTGGTGGGTT
ChIP-p3-F	GTGTTGGAACCACCAGTGAC
ChIP-p3-R	CTGTCTATGGGTGGTGGGTT
ChIP-IGS-F	ACAGACCCACCATCCCCCAA
ChIP-IGS-R	GCTCTCTGTCTATGGGTGGT
ChIP-18S-F	ATCTGTCAATCCTTTCCG
ChIP-18S-R	GGGGAGTATGGTTGCAA
ChIP-28S-F	GAGTAGTGGTATTTCACC
ChIP-28S-R	GACGCGCATGAATGGATG
gapdh-F	GCCGTGGTGCCAGTCAGAA
gapdh-R	GGTGCTCCGTGTATCCCAGAAT

Supplementary Materials and methods

1. Key resources table

REAGENT or RESOURCE	SOURCE	IDENTIFIER
**Antibodies**		
Rabbit polyclonal Rpa2 antibody	Huang Lab (Mu et al., 2016)	
Mouse monoclonal PCNA antibody	Sigma-Aldrich	Cat# P8825, RRID:AB_477413
Rabbit polyclonal Fen1 antibody	Abcam	Cat# ab17994, RRID:AB_444168
Rabbit polyclonal Chk2 antibody	AnaSpec	Cat# 55435, RRID:AB_1963092
Rabbit polyclonal Rad51 antibody	AnaSpec	Cat# 55838
Mouse monoclonal Bhmt antibody	Huabio	Cat# HAMH0741
Mouse monoclonal Fibrillarin antibody	Abcam	Cat# ab4566, RRID:AB_304523
Rat monoclonal Brdu antibody	AbD Serotec	Cat# OBT0030, RRID:AB_609568
Mouse monoclonal GFP antibody	Santa Cruz	Cat# sc-9996, RRID:AB_627695
Mouse monoclonal Flag antibody	Sigma-Aldrich	Cat# F1804, RRID:AB_262044
Mouse monoclonal P53 antibody	Huabio	Cat# HACC0511
Mouse monoclonal Ttf1 antibody	Huabio	Cat# HAMJ1114
Mouse monoclonal Bms1l antibody	Huabio	Cat# HAMH0402
Rabbit polyclonal Bms1l antibody	Huabio	Cat# HAPH0412
Rabbit polyclonal Rcl1 antibody	Huabio	Cat# HAPI0134
Rabbit polyclonal CyclinE1 antibody	Huabio	Cat# HAPJ0149
Rabbit polyclonal HA antibody	Sigma-Aldrich	Cat# H6908; RRID: I2149
Mouse monoclonal GAPDH antibody	Huabio	Cat#M1211-1
Mouse monoclonal α-Tubulin antibody	Beyotime	Cat# AT819-1
Rabbit polyclonal β-Actin antibody	Huabio	Cat# R1207-1
Rabbit Ttf1 antibody	Millipore	Cat# 32160702
Rabbit Chk2 antibody	Abcam	Cat#47433
Rabbit phosph-Chk2(Thr68) antibody	CST	Cat#2197
**Chemicals**		
Anti-Digoxigenin-AP Fab fragments	Roche	Cat# 11093274910
Anti-HA-Tag mAb (Agarose conjugated)	Abmart	Cat# M20013
Anti-Flag-Tag mAb (Agarose conjugated)	Yeason	Cat# 710040
HA-Tag peptide	Huabio	Customize
Flag-Tag peptide	Yeason	Cat#20572ES11

Protein A/G agarose beads	Beyotime	Cat# P2019
Complete, EDTA-free	Roche	Cat# 16829800
OCT	Leica	Cat# 090913
BrdU	Sangon biotech	Cat# E607203
EdU	Invitrogen	Cat# A10044
**Critical commercial assays**		
EasySee Western Blot Kit	Trans	Cat# DW101-02
Maxi ECL Substrate	Sunky	Cat# 61804
PCR Cleanup Kit	Axygen	Cat# 156
Plasmid Miniprep Kit	Axygen	Cat# 155
**Experimental models: cell lines**		
Human: HEK293T cells	ATCC	Cat# CRL-3216; RRID: CVCL_0063
**Experimental models: zebrafish**		
Zebrafish: AB	Singapore	N/A
Zebrafish: *bms1l*^{sq163}	This paper	N/A
Zebrafish: *bms1l*^{zju1}	This paper	N/A
Zebrafish: *rcl1*$^{-/-}$	This paper	N/A
Zebrafish: *p53*^{M214K}	(Berghmans et al., 2005)	N/A
Zebrafish: *Tg(lfabp:RFP; elaA:EGFP)*	(Wan et al., 2006)	N/A
**Recombinant DNA**		
pCS2^{+}*-HA-Bms1l-WT*	This paper	N/A
pCS2^{+}*-HA-Bms1l-163*	This paper	N/A
pCS2^{+}*-HA-Bms1l-X*	This paper	N/A
pCS2^{+}*-Flag-Ttf1a*	This paper	N/A
pCS2^{+}*-Flag-Ttf1b*	This paper	N/A
pCS2^{+}*-Flag*	This paper	N/A
pCS2^{+}*-TS-EGFP*	This paper	N/A

2. Materials and methods

Zebrafish lines and maintenance

The zebrafish AB line was used as the wild type (WT) in this work. Fish were raised and maintained according to standard procedures (Lo et al., 2003). The *bms1l*^{sq163} mutant line was obtained by screening for small liver mutants after ENU mutagenesis (Wang et al., 2012). The *bms1l*^{zju1} mutant line was generated by CRISPR-Cas9 technology using a gRNA (GCATGGCAAAAACCTTCCATCGGTTAG) specifically targeting the junction between exon2 and intron2 of the *bms1l* gene. The *rcl1*$^{-/-}$ mutant harbors a 16bp insertion and one base substitution

in the first exon that create an early stop codon in the *rcl1* ORF containing the N-terminal 36 amino acids (aa) of Rcl1 plus 18 aa derived from the ORF shift. The *p53*^{M214K} mutant was obtained from Thomas Look (Harvard Medical School, USA) (Berghmans et al., 2005). Transgenic line *Tg(lfabp:RFP; elaA:EGFP)* was obtained from Zhiyuan Gong (National University of Singapore, Singapore) (Wan et al., 2006).

Cell lines and cell transfection

Human 293T cells were maintained in DMEM (high glucose, GIBCO), supplemented with 10% newborn calf serum (NBCS, GIBCO). Plasmids were transfected into the cells using Lipofectamine 2000 (Invitrogen) transfection reagent according to the manufacturer’s recommendations.

Plasmid construction

The HA-tag was fused in-frame to the 3’-end of the full-length cDNA of zebrafish *bms1l-WT*, *bms1l-sq163* and *bms1l-X*, respectively, by reverse transcription coupled with polymerase chain reaction (RT-PCR) using the HA-Bms1l-F and HA-Bms1l-R primer pair (Supplementary Table S7). The *bms1l-WT-HA*, *bms1l-163-HA* and *bms1l-X-HA* products were cloned into the *pCS2*$^{+}$ expression vector, respectively. The full-length cDNA of zebrafish WT *ttf1a* and *ttf1b* was obtained through RT-PCR using a pair of primers Flag-Ttf1-F and Flag-Ttf1-R shared by *ttf1a* and *ttf1b* (Supplementary Table S7), respectively. The *pCS2*$^{+}$*-Flag-Ttf1a* and *pCS2*$^{+}$*-Flag-Ttf1b* plasmids were constructed by cloning the *ttf1a* and *ttf1b* cDNA into the *pCS2*$^{+}$*-Flag* expression vector, respectively. The *pCS2*$^{+}$*-Flag* construct was obtained from Qiang Wang (Institute of Zoology, Chinese Academy of Sciences, China).

Northern blotting, 28S/18S ratio quantification and real-time quantitative PCR (qPCR)

Total RNAs were extracted from embryos using Trizol Reagent (Invitrogen, 15596-026). The digoxigenin (DIG)-labeled 5’ETS and ITS1 DNA probes were as described previously (Tao et al., 2013). RNA gel blot hybridization was performed as described previously (Chen et al., 2005). The qPCR values were normalized against *GAPDH* and expressed as fold change of expression. The values plotted represent mean ± SEM. For 28S/18S ratio analysis, total RNAs were analyzed by Aglient Bioanalysis 2100 (Aglient, USA) according to manufacturer's instructions. The qPCR was performed as described previously (Tao et al., 2013). Primer pairs used for qPCR analysis of different genes were listed in Supplementary Table S8.

Whole-mount in situ hybridization (WISH)

Digoxigenin (DIG)-labeled RNA probes (Roche DIG RNA Labeling mix 11277073901) were

used in WISH. WISH was performed as described. *fabp10a*, *ifabp* and *trypsin* gene fragments were respectively cloned into the pGEM-T vector containing T7, T3 or Sp6 promoter sequence, and these plasmids were used as templates to generate the corresponding RNA probes via *in vitro* transcription for WISH (Gao et al., 2019).

Protein analysis, co-immunoprecipitation (Co-IP) and antibodies

Zebrafish embryos were disassociated by passing it through a 21G needle several times in PBS. After centrifugation, the supernatant was discarded and protein was extracted in SDS sample buffer (63mM Tris-HCl pH6.8, 10% Glycerol, 5% β-mercaptoethanol, 3.5% SDS) by heating at 100 ℃ for 7 minutes. Western blot analysis was performed as described (Wang et al., 2016).

For the Co-IP experiment, total protein was extracted from cultured cells through the following procedure. Cells were collected into a 1.5ml tube, washed twice in pre-chilled PBS buffer and then sonicated (on 10s, off 15s, amplitude 30%, 5mins, Branson) in IP lysis buffer (P0013J, Beyotime). 10% of the lysis product was kept as input. 20 μl of agarose conjugated antibody beads were added into the remaining lysate and the mixture was incubated at 4 ℃ overnight. The next day, the beads were collected by a brief spinning, washed in PBST (PBS+0.1% Tween20) five times and heated at 100 ℃ for 10 minutes in Lane Marker Sample Buffer (26149, Thermo Scientific). After centrifugation, the supernatant was collected as the protein sample. Protein electrophoresis and western blots were performed according to the instructions provided by the manufacturers.

Primary antibodies used in this work included antibodies against PCNA (P8825, Sigma), Fen1 (ab17994, abcam), Chk2 (55435, Anaspec), Rad51 (55838, Anaspec), Fibrillarin (ab4566, abcam), BrdU (OBT0030, AbD sero Tec), GFP (sc-9996, Santa Cruz), HA (AH158, Beyotime), Flag (F1804, Sigma), β-Actin (R1207-1, Huabio), GAPDH (M1211-1, Huabio), Tubulin (AT819, Beyotime). Monoclonal antibodies against P53, Ttf1, Bms1l, Bhmt and rabbit polyclonal antibodies against Rcl1, CyclinE1 and Bms1l were generated by Hangzhou HuaAn Biotechnology Company. Rabbit polyclonal antibody against Rpa2 was obtained from Dr Jun Huang (Life Sciences Institute, Zhejiang University, China).

ttf1 morpholino (ttf1-MO) and the specificity of the Ttf1 monoclonal antibody

Morpholino was purchased from Gene Tools (Philomath, USA). The *ttf1* morpholino (*ttf1*-MO) (5'-ACGATGGATGAGATGCTGTCAGATT-3') was designed to target the common sequences of *ttf1a* and *ttf1b* transcripts in the region around the translation start codon (TS) ATG. To examine the efficiency of *ttf1*-MO, a primer (i.e EGFP-TS-F) was designed by fusing the *ttf1a/ttf1b* TS sequence upstream to the 5'-end of the *EGFP* gene (Supplementary Table S7). The EGFP-TS-F and EGFP-TS-R primer pair (Supplementary Table S7) was used to amplify the *EGFP* gene to generate

the *pCS2*[+]*-TS-EGFP* plasmid. *ttf1*-MO (0.6 pmol) and pCS2+-TS-EGFP plasmid (150 pg) were injected into one-cell stage embryos. Injected embryos were subjected to total protein extraction for western blot analysis and GFP fluorescence observation under a fluorescence microscope at 8 hours post-injection. We found that *ttf1*-MO successfully knocked down the expression of the *Egfp* reporter gene (Supplementary Figure S7F and G).

The Ttf1 monoclonal antibody could detect both Ttf1a and Ttf1b overexpressed in the cultured 293T cells (Supplementary Figure S7E). We confirmed the identity of the endogenous Ttf1 protein in western blot by comparing protein patterns between WT and *ttf1*-MO-injected embryos at 3dpf (Supplementary Figure S7H).

Cryo-sectioning and immunostaining

For cryo-sectioning, embryos were fixed in 4% PFA for 2 hours. The samples were embedded in 1.5% agarose/30% sucrose, followed by permeation in 30% sucrose overnight. The next day, samples were further embedded by OCT (090913, Leica) in suitable molds in -80℃ ethanol. Sections of 10μm thickness were mounted onto coated glass slides. Frozen sections were kept at -80℃ until use.

Before immunostaining, the frozen slides were first kept at room temperature for a few minutes. When an antigen retrieval treatment was required, the slides were immersed in retrieval buffer (10mM sodium citrate, pH 6.0) and subjected to mid-low heat in a microwave oven for about 5 minutes. The slides were then washed in PBST buffer (PBS plus 0.2% TritonX-100) for 3×5 minutes. To reduce non-specific binding, the slides were first blocked in 10% goat serum diluted in PBST for about 40 minutes and then incubated with primary antibody diluted in blocking solution overnight at 4℃. The next day, slides were washed in PBST for 3×5 minutes. Secondary antibodies conjugated with fluorescence (1:400 diluted in blocking buffer) and DAPI (1:1,000 diluted in blocking buffer) were then added to the slides and incubated at room temperature (~24℃) for 2 hours. After washing with PBST, samples were mounted by a drop of glycerol and covered by coverslips and then sealed by nail oil. All immunofluorescence staining images were taken under an Olympus BX61WI confocal microscope.

EdU and BrdU incorporation assay

For the EdU (5-ethynyl-2′-deoxyuridine) single-incorporation assay, EdU (1nl, 10 mM) was injected into the heart of 4dpf- or 5dpf-old embryos. The injected embryos were incubated at 28.5℃ till the desired time point for fixation in 4% PFA for 2 hours and were then subjected to cryo-sectioning. Incorporated EdU was detected by Alexa Fluor 488 Azide (Life Technologies, A10266).

For the EdU and BrdU double-incorporation assay, 10 mM EdU was injected into the heart of 4dpf-old embryos. After incubation at 28.5 ℃ for 5 hours, BrdU (5-bromo-2-deoxy-uridine, 1nl, 10 mM) was injected into the heart of embryos. After incubation at 28.5 ℃ for 7 hours, the injected embryos were fixed in 4% PFA for 2 hours prior to cryo-sectioning. Cryosections were incubated in 2N HCl for 40 minutes at 37°C to exposure the incorporated BrdU, followed by immunostaining of BrdU and detection of EdU.

All immunofluorescence staining images were taken under an Olympus BX61WI confocal microscope.

Flow cytometry analysis

Approximately 100 *Tg(lfabp:RFP)* zebrafish embryos were collected and fixed with 0.5% PFA at room temperature for 2 hours. Livers were then dissected under a fluorescence microscope, followed by treatment with 0.1% trypsin at 37 ℃ for 30 minutes. Cells were then gently pipetted and washed in PBS twice by centrifugation at 1800g for 5 minutes and then permeated with 70% ethanol at 4 ℃ for 2 hours. To confirm the hepatocyte identity, cells were washed and resuspended by PBS, then incubated with the Bhmt antibody (1:2000) at 4 ℃ overnight, followed by incubation with Alexa Fluor 488 anti-rabbit secondary antibody (1:500) at 4 ℃ for 4 hours to specifically label the hepatocytes. For DNA content detection, cells were washed by PBS and resuspended in 300ul PBS, then incubated with Propidium Iodide (50μg/ml) at room temperature for 30 minutes and subjected to flow cytometry analysis of the cell cycle on BD FACS Calibur flow cytometer (Guan et al., 2016).

Immuno-transmission electron microscopy (Immuno-TEM) detection of Rpa2

Embryos were first fixed in 2.5% glutaraldehyde (0.1M PBS, pH 7.2-7.4) at room temperature for 1 hour, followed by three times washes in PBS for 10 minutes each. Livers were then dissected and fixed in the similar fixative at 4 ℃ overnight, followed by four times washes in 4% sucrose/0.1M PBS at 4 ℃ for 15 minutes each and further incubation in 4% sucrose/0.1M glycine/0.1M PBS at 4 ℃ for 30 minutes. Dehydration was carried out in increasing concentrations of methanol (30% and 50% methanol at 4 ℃, 70%, 80%, 95% methanol at -20 ℃, 100% methanol at -20 ℃ three times for 15 minutes each). Gradient permeation was performed in 100% methanol/Lowicryl K4M (1:1) at -20 ℃ for 1 hour, 100% methanol/K4M (1:3) at -20 ℃ for 1 hour and K4M resin at -20 ℃ overnight. Samples were then transferred to a new PCR tube with an equal volume (200ml) of newly prepared K4M. Polymerization was carried out at -20 ℃ for 72 hours, followed by UV illumination at room temperature for 48 hours.

After sectioning (100nm), grids were rinsed with distilled water, followed by incubation in

blocking solution (1% BSA, 0.02% PEG20000, 100mM NaCl, 1% NaN_3, 0.22μm strainer filtered) for 5 minutes. Then grids were incubated with RPA2 antibody (1:50), at 4 ℃ overnight, followed by six times washes in 0.01M PBS for 2 minutes each. Secondary antibody incubation was performed with Protein-A gold (1:100) (Center of Electron Microscopy, Zhejiang University) at room temperature for 2 hours, followed by similar washing conditions and additional four times washes with distilled water for 2 minutes each. The sections were then stained with 3% uranyl acetate for 5 minutes and lead citrate (1.33g lead nitrate, 1.76g sodium citrate, 8mmol NaOH in 50ml ddH_2O) for 3 minutes. Grids were observed with a Hitachi transmission electron microscope (H-7650) at 80kV and 8000~80000X magnification.

ttf1a/ttf1b expression analysis

Total RNA was extracted from 2dpf-, 3dpf-, 4dpf- and 5dpf-old WT embryos for generating cDNA by reverse transcription (Invitrogen, 28025-021). PCR products were obtained by using a pair of primers (F: CGACTCATTAAAGCGATGTATGA, R: CTATTGATTAAAGCTGTTGTTCT) perfectly matching both *ttf1a* and *ttf1b* sequences and then cloned into the pGEM-T vector. 96 individual *E coli* colonies were randomly picked for DNA sequencing to identify clones corresponding to *ttf1a* or *ttf1b* based on single nucleotide polymorphisms (SNPs) between *ttf1a* and *ttf1b.*

ChIP-seq and ChIP-qPCR

The ChIP-seq protocol described here is based on previous studies with several modifications for its use in zebrafish embryonic stages. Zebrafish embryos at 3dpf were deyolked in PBS by shivering 5-10 times using 21G (0.8mm) needle and 2ml syringe. The cell pellet was suspended in 1ml NIM buffer (0.25mM sucrose, 25mM KCl, 10mM Tris-HCl pH 7.4, 5mM $MgCl_2$) and homogenized. Formaldehyde was added to a final concentration of 1% at room temperature for 20 minutes. Glycine was then added to a final concentration of 125mM to quench the reaction. The nuclei pellet was washed in 1ml NIM buffer. The pellet was resuspended in 275μl CHIP lysis buffer (50mM Tris-HCl pH 8.1, 10mM EDTA, 1% SDS) followed by incubation in ice for 20 minutes. 275μl CHIP dilution buffer (0.01% SDS, 1.1% Triton X-100, 1.2mM EDTA, 16.7mM Tris-HCl pH8.1, 167mM NaCl) was added for sonication (on 5s, off 20s, amplitude 30%, 16mins, Branson) to obtain the DNA fragment between 100~500bp. The supernatant was taken for immunoprecipitation by adding 1500ul CHIP dilution buffer and 20μg Ttf1 antibody and the mixture was incubated overnight at 4 ℃. Mouse IgG was used as a negative control through the same procedure. The next day, protein A/G agarose beads (P2019, Beyotime) was added and incubated at 4 ℃ for 4 hours, then the pellet was washed consecutively with each of following

solution: low salt wash buffer (Tris-HCl 20mM pH 8.1, 2mM EDTA, 150mM NaCl, 0.1% SDS, 1% Triton), high salt wash buffer (Tris-HCl 20mM pH 8.1, 2mM EDTA, 500mM NaCl, 0.1% SDS, 1% Triton), LiCl wash buffer (Tris-HCl 10mM pH 8.1, 0.25M LiCl, 1% NP-40, 1% deoxycholic acid, 1mM EDTA), TE buffer (Tris-HCl 10mM pH 8.1, 1mM EDTA). Freshly prepared elution buffer (100μl 1M $NaHCO_3$, 100μl 10% SDS, 800μl H_2O) containing RNaseA was added to a final concentration of 0.33μg/μl, and the mixture was incubated at 65 ℃ for 4 hours. 0.5mg/ml proteinase K was added and incubated at 60 ℃ for 2 hours. The DNA was sent to ANNOROAD (Beijing, China) for sequencing after precipitating and purify.

ChIP-qPCR primers were designed based on CHIP DNA peaks and listed in Supplementary Table S8. qPCR was performed as described previously (Tao et al.,2013).

Assay for Ttf1a binding to peak-3

Plasmids of *Bms1l-WT-HA* (1μg), *Bms1l-163-HA* (1.6μg), *Bms1l-X-HA* (1.6μg), *Flag-Ttf1a* (0.5μg), *HA-pCS2+* (1μg) were transfected into 293T cell line, respectively. 48h post transfection, cultured cells were harvested and rinsed twice with PBS, followed by centrifugation at 3000g at 4°C. The cells were resuspended in the extraction buffer (10mM PBS, 5mM KCl, 1mM Dithiothreitol (DTT), 5mM $MgCl_2$, pH 7.4) and extracted by subjecting to liquid nitrogen multi-gelation three times. The cells were then centrifuged at 12000*g* 4°C for 10 min, and the supernatant was collected. To purify the protein, 20μl Anti-Flag-Tag affinity agarose beads (710040, Yeason) or Anti-HA-Tag affinity agarose beads (314080, Abmart) were added and incubated for 4~6h at 4°C. Beads were washed with extraction buffer for 3 times. 1mg/ml Flag peptide or HA peptide was used to elute the protein from the beads.

For DNA-binding assay, purified Flag-Ttf1a protein was mixed with purified Bms1l-WT-HA, Bms1l-163-HA, Bms1l-X-HA, HA-pCS2+ and 5fmol peak-3 DNA fragment with or without GTP (Thermol Scientific) and was incubated at 28°C for 1h. 20μl Anti-Flag-Tag affinity agarose beads were then added and incubated at 28°C for 1h. The beads were pelleted by centrifugation at 1000g/min for 1 min and the pellet was washed sequentially with each of following solution: low salt wash buffer **(**Tris-HCl 20mM, pH 8.1, 2mM EDTA, 150mM NaCl, 0.1% SDS, 1% Triton), high salt wash buffer (Tris-HCl 20mM, pH 8.1, 2mM EDTA, 500mM NaCl, 0.1% SDS, 1% Triton), LiCl wash buffer (Tris-HCl 10mM, pH 8.1, 0.25M LiCl, 1% NP-40, 1% deoxycholic acid, 1mM EDTA), and TE buffer (Tris-HCl 10mM, pH 8.1, 1mM EDTA). The pellet in TE buffer was incubated in a heater at 80°C for 10 min, and was centrifuged at 12000*g* 4°C for 1min. The supernatant was collected for qPCR analysis, and the pellet was used as the protein sample for western analysis.

GTPase activity assay

The GTPase activity assay is based on checking the inorganic phosphate (Pi) level released from a phosphorylated substrate. This assay is performed under standard procedure of PiColorLock Phosphate Detection System (303-0030, expedeon). Using purified Bms1l-WT-HA, Bms1l-163-HA, Bms1l-X-HA with GTP (Thermol Scientific) to start the enzyme reaction for 5mins and 60mins. The absorbance values at a wavelength between 590nm and 650nm were recorded. EDTA was used to stop the enzyme activity.

Human BMS1 knockdown analysis

HeLa cell were cultured in DMEM (10% fetal bovine serum and 1% penicillin and streptavidin) maintained at 37 ℃ in 5% CO_2. Two siRNAs (siBMS1#3 and siBMS1#6) specifically targeting human BMS1 and a control siRNA (Supplementary Table S5) were purchase from Ribobio. siRNAs were (40nM) transfected twice using Lipofectamine RNAiMAX (Invitrogen) according to the instruction. Expression vectors were transfected with Liposomal Transfection Reagent (Yeason). For protein sampling, cell were lysed with NETN buffer (containing 1μg/ml aprotinin and leupeptin) and incubated on ice for 15mins. After centrifugation, the supernatants were used to performing immunoblotting with standard procedures. Antibodies against human BMS1 and TTF1 were used to detect endogenous BMS1 and TTF1 in HeLa cells, respectively. For BrdU/PI double labeling and flow cytometry analysis, 20μM BrdU was added into medium for 1hr before harvest the cell. Washed the cell with PBS, and fixed with pre-colded 70% ethanol overnight. Using 2.5M HCl to denature the DNA double helix. After washing 3 times with PBS, cell were incubate with mouse anti-BrdU antibody (1:100, Roche) in blocking buffer (PBS+0.1%Triton X-100+5%BSA) for 12hrs. Washing 3 times with blocking buffer adding 300mM NaCl. Incubate with FITC-conjugated goat anti-mouse IgG (1:100) for 4hrs. Washing 3 times with blocking buffer adding 300mM NaCl. Cells were resuspended in PBS with propidium iodide (PI) (50μg/ml) and 200μg/ml RNase A at room temperature for 30 minutes and subjected to flow cytometry analysis on BD FACS Calibur flow cytometer.

For RPA2 immunofluorescence staining, cell were cultured on coverslips. Incubate with 0.5% Triton X-100 in PBS for 5mins at room temperature, followed by fixed with 3% paraformaldehyde for 10mins at room temperature. After washing with PBS, incubate cells with primary antibodies for 20mins at room temperature. Washing 3 times with PBS, the coverslips were incubate with secondary antibodies and DAPI for another 20mins at room temperature. Images were taken under an Olympus BX61WI confocal microscope. RPA2 signals were captured using ImageJ software.

Plasmids expressing Flag-tagged human BMS1 (Flag-BMS1^R) and BMS1-163 (Flag-BMS1-163^R) were respectively constructed via site-guided mutagenesis without altering the

BMS1 coding sequence using primers Flag-BMS1R-R and Flag-BMS1R-F (Supplementary Table S5).

Quantification and statistical analysis

For comparing 28S/18S rRNA ratios, the peak values of 18S and 28S rRNA were calculated by Aglient Bioanalysis 2100 (Aglient, USA) from three independent samples for each genotype.

qPCRs were performed on a CFX96TM Real-Time System (Bio-Rad) cycler and data were normalized against GAPDH.

For comparing the number of nucleoli between WT and *bms1l*^{sq163}, hepatocytes and pancreatic cells were categorized into three groups based on the number of nucleoli (1, 2 or ≥3) within each cell. For comparing the size of nucleolus between WT and *bms1l*^{zju1} hepatocytes, the areas of nucleolus and nucleus in each cell were calculated by Photoshop for analyzing the area ratio of nucleolus to nucleus.

For comparing the ratio of PCNA, EdU, BrdU, Rpa2, Ttf1 or pH3 signal positive cells between WT and *bms1l* mutants, the number of signal-positive cells, double-labeled cells and total cells in liver or gut were counted and compared. For comparing the ratio of PCNA or EdU signal positive cells in neural tube between WT and *bms1l* mutants, the areas of signal-positive cells and total cells were measured and compared.

For comparing the Ttf1 signal intensity in WT, *bms1l* or *rcl1* mutant, the intensity of Ttf1 and DAPI were calculated by FV10-ASW 3.0 viewer, respectively, for analyzing the ratio of intensity of Ttf1 to DAPI.

For comparing the DNA content in hepatocytes between *bms1l*^{sq163} and its siblings, the peak value of 2n, 4n and polyploidy were calculated by BD FACS Calibur flow cytometer from three independent samples for each genotype.

For the immuno-TEM analysis of Rpa2 in WT and *bms1l*^{sq163} mutant hepatocytes, the number of gold particles was counted and the areas of nucleolus and nucleoplasm were measured based on pixels for calculating the ratio of gold particles spots to area.

For comparing the liver and pancreas development among the siblings, *p53*^{M214K} single mutant, *bms1l*^{sq163} single mutant and *bms1l*^{sq163} *p53*^{M214K} double mutant, the areas of liver, pancreas and yolk were measured, respectively, for analyzing the area ratio of liver or pancreas to yolk.

For statistic analysis, comparisons were made using the Student's *t*-test assuming a two-tailed distribution, with significance being defined as $p < 0.05$ (*), $p < 0.01$ (**) and $p < 0.001$ (***), no significance (NS).

References

Berghmans, S., Murphey, R.D., Wienholds, E., Neuberg, D., Kutok, J.L., Fletcher, C.D., Morris, J.P., Liu, T.X., Schulte-Merker, S., and Kanki, J.P., et al.(2005). tp53 mutant zebrafish develop malignant peripheral nerve sheath tumors. Proc Natl Acad Sci U S A 102, 407-412.

Chen, J., Ruan, H., Ng, S.M., Gao, C., Soo, H.M., Wu, W., Zhang, Z., Wen, Z., Lane, D.P., and Peng, J.(2005). Loss of function of def selectively up-regulates Delta113p53 expression to arrest expansion growth of digestive organs in zebrafish. Genes Dev 19, 2900-2911.

Gao, C., Huang, W., Gao, Y., Lo, L.J., Luo, L., Huang, H., Chen, J., and Peng, J.(2019). Zebrafish hhex-null mutant develops an intrahepatic intestinal tube due to de-repression of cdx1b and pdx1. J MOL CELL BIOL 11, 448-462.

Guan, Y., Huang, D., Chen, F., Gao, C., Tao, T., Shi, H., Zhao, S., Liao, Z., Lo, L.J., and Wang, Y., et al.(2016). Phosphorylation of Def Regulates Nucleolar p53 Turnover and Cell Cycle Progression through Def Recruitment of Calpain3. PLOS BIOL 14, e1002555.

Lo, J., Lee, S., Xu, M., Liu, F., Ruan, H., Eun, A., He, Y., Ma, W., Wang, W., and Wen, Z., et al.(2003). 15000 unique zebrafish EST clusters and their future use in microarray for profiling gene expression patterns during embryogenesis. GENOME RES 13, 455-466.

Mu, Y., Lou, J., Srivastava, M., Zhao, B., Feng, X.H., Liu, T., Chen, J., and Huang, J.(2016). SLFN11 inhibits checkpoint maintenance and homologous recombination repair. EMBO REP 17, 94-109.

Tao, T., Shi, H., Huang, D., and Peng, J.(2013). Def functions as a cell autonomous factor in organogenesis of digestive organs in zebrafish. PLOS ONE 8, e58858.

Wan, H., Korzh, S., Li, Z., Mudumana, S.P., Korzh, V., Jiang, Y.J., Lin, S., and Gong, Z.(2006). Analyses of pancreas development by generation of gfp transgenic zebrafish using an exocrine pancreas-specific elastaseA gene promoter. EXP CELL RES 312, 1526-1539.

Wang, Y., Luo, Y., Hong, Y., Peng, J., and Lo, L.(2012). Ribosome biogenesis factor Bms1-like is essential for liver development in zebrafish. J GENET GENOMICS 39, 451-462.

Wang, Y., Zhu, Q., Huang, L., Zhu, Y., Chen, J., Peng, J., and Lo, L.J.(2016). Interaction between Bms1 and Rcl1, two ribosome biogenesis factors, is evolutionally conserved in zebrafish and human. J GENET GENOMICS 43, 467-469.
